# Analectic

**Published:** 1831

**Authors:** 


### MISCELLANEOUS INTELLIGENCE.

### ANALECTIC, ANALYTICAL, AND ORIGINAL.

### I. ANALECTIC.

1. *Experiments on Pulmonary Absorption and Exhalation.*—M. Collard de Martigny, has published in the 'Journal Complementaire,' for May and August last, an account of some interesting set of experiments, undertaken for the purpose of determining some of the disputed points in the chemical physiology of respiration. Not having received that journal, we are indebted to the Edinburgh Medical and Surgical Journal, for a summary of his principal results. They are the following:

1. According to the doctrine of Lagrange, which is a modification of the original theory of Lavoisier, oxygen gas is absorbed *in substance* by the venous blood, in passing through the lungs, and does not unite with oxygen to form carbonic acid, till it has proceeded with the arterial blood to the capillaries. This opinion rests merely on some imperfect experiments by Girtanner, who thought he discovered oxygen in arterial blood. It is singular that these experiments should not have been repeated till now, as they obviously, if correct, lead to a very precise and important conclusion. On trying them, M. Collard de Martigny procured results decidedly negative. Having filled a tube, thirty-six inches long, with mercury, and reversed it so as to produce a barometric vacuum, he admitted about an inch of fresh arterial blood, from the crural artery of a living dog, and left the apparatus at rest for an hour and a half. At this time, the mercury having descended considerably, the gas which had been developed. was transferred into a graduated tube, and acted on by caustic potass, The whole of it was entirely absorbed, and consequently consisted of carbonic acid only.

2. The more prevalent doctrine at present is, that the oxygen which is absorbed by the blood in its passage through the lungs, *unites with* the blood; that carbonic acid is formed in the capillaries by the various processes of nutrition and secretion; and that the carbonic acid thus formed, is given off in the lungs, by a process of exhalation and secretion, independently of the presence of oxygen. This doctrine rests mainly on an experiment first performed by Girtanner, and afterwards more carefully by Edwards—where a frog, being made to breathe hydrogen alone, gave off, nevertheless, more than its own bulk of carbonic acid, in the course of a few hours. This result, if the mode of experimenting is free from fallacy, is decisive of the question. It proves, that the carbonic acid given off during respiration, is not formed in the lungs by the union of oxygen with the carbon of the venous blood in its passage through the pulmonary circulation, but arrives with the venous blood in the lungs ready-formed, and is, in short, the product of the various functions of the capillaries. But M. Collard D. Martigny considers, that even the method of experimenting pursued by Dr. Edwards, is liable to fallacy. On the one hand, hydrogen and carbonic acid are so different in density, that they mingle slowly, and in consequence, an analysis of a portion of the mixture does not represent the composition of the whole mass. And on the other hand, according to a law in physics, the tissues of the body, being impregnated with carbonic acid, must give out that gas when immersed in an atmosphere of any other gas. To obviate the former fallacy, he substituted azote for hydrogen; and to do away as much as possible with the latter, he withdrew and analyzed the air in which the animal was confined, once every hour or every two hours, and replaced it by pure azote—so that in this way he could ascertain whether carbonic acid was given out only at first, and therefore arose merely from the displacement of the gas diffused through the textures, or continued throughout the whole duration of the experiment, and consequently arose from the secretion of the gas by the lungs. In every experiment, he found carbonic acid given out in considerable quantity, as Edwards also invariably remarked. In an interval, varying from seven and a half to nine hours, he procured, in seven different experiments, from 2 1-2 to 2 3-4 centilitres, or between 1 1 2 and 1 3-4 cubic inch of carbonic acid. In every instance he found nearly twice as much gas formed during the first period, as during any subsequent period, which he attributes to the displacement of carbonic acid in the textures by the azote. But after the first period, the quantity formed in every equal period of time, was nearly the same, till the animal began to become torpid and the respiration to languish, when the formation of carbonic acid rapidly decreased. Hence he concludes, that after the first period, the carbonic acid is derived solely from the blood in the lungs.

The exhalation of carbonic acid from the lungs, then, is independent of oxygen being supplied to it. The presumption must consequently be, that it is secreted or excreted by the blood in the lungs. But in order to establish this doctrine satisfactorily, it is necessary to show that the blood in passing through the lungs, really loses carbonic acid—a point which no one before M. Collard de Martigny has endeavored to ascertain. He has proved it, however, as he conceives, by a comparative examination of the arterial and venous blood of the same animal. When each was collected directly from the blood vessels in a barometric vacuum, as formerly described, he found that venous blood almost always gave out twice, and on one occasion, thrice as much carbonic acid as the arterial blood. But when respiration was suspended by exhausting the lungs, and tying the trachea, the arterial blood was found to contain as much carbonic acid as the blood in the veins.

3. Physiologists have differed with one another as to the question, whether the carbonic acid gas given out in the lungs is equivalent to the oxygen absorbed by them; but, on the whole, the prevailing opinion is, that more oxygen is absorbed than is accounted for by the carbonic acid exhaled. M. Collard de Martigny arrives at the same conclusion by a series of experiments, apparently more free from fallacy than any previously made. The chief difficulty is to avoid the fallacy arising from the probability of the air in the lungs of the animal at the beginning of the experiment not corresponding in quantity with what remains at the end. The author got rid, as he conceives, of this difficulty, by not confining the animal in the jar of air to be breathed, but by fixing a tube in its trachea, exhausting the air in its lungs, then establishing a communication by means of the tube between the lungs and the jar, and, at the end of the experiment, expelling the residual air of the lungs into the jar by strong pressure of the chest. He has related the particulars of nine experiments of this kind, of which eight were performed with the rabbit, and one with the Guinea-pig. In four of them, the quantity of air breathed was four *litres*, or 244 English cubic inches; in four others, it was three *litres* and a half, or 213 cubic inches, in one it was 183 cubic inches; and the duration of the experiment varied from nine to fifteen minutes. In the largest quantity of air, the quantity of oxygen was 51.2 cubic inches. Of this there remained unaccounted for by the residue of oxygen, together with the carbonic acid evolved, 6.5, 16.3, 16.9, and 16.9 cubic inches in four different experiments. In the four experiments with 213 cubic inches of air, the total oxygen being 44.8 cubic inches, there remained unaccounted for, in like manner, 2.3, 4.7, 7.1, 10.4, and 18.4 cubic inches. In the experiment with 183 cubic inches, where the oxygen was 38-4, the quantity unaccounted for was 4.7 cubic inches.* In all these experiments, then, it is clear, that a large, though variable quantity of oxygen gas disappears—a larger quantity is absorbed than is given off in the form of carbonic acid. But we must observe that

*The particulars are contained in the following tables, which are carefully calculated from the original tables of the author.

| Oxygen in Air before Respiration. | Oxygen remaining. | Carbonic Acid. | Oxygen disappeared. |
|---|---|---|---|
| 1. 51.2 | 22 | 12.3 | 16.9 |
| 2. | 27.1 | 17.6 | 6.5 |
| 3. | 22.6 | 11.8 | 16.9 |
| 4. | 20.3 | 14.6 | 16.3 |
| 5. 448. | 17.4 | 17.0 | 10.4 |
| 6. | 14.4 | 12.0 | 18.4 |
| 7. | 19.7 | 22.7 | 2.3 |
| 8. | 18.7 | 19.0 | 7.1 |
| 9. 38.4 | 15.3 | 18.4 | 4.7 |

M. Collard de Martigny commits a serious oversight in supposing that his method of experimenting is free of fallacy, or represents natural respiration. On the contrary, after the first inspiration, the animal breathes an atmosphere considerably impregnated with carbonic acid gas; consequently the blood absorbs the gas, which it is very well known to do when a moderate proportion of carbonic acid in the air is presented to it; and in this way the apparent disappearance of oxygen may be sufficiently accounted for.

4. Another point of dispute among physiologists who have occupied themselves with this subject, is whether any azote is given off or absorbed. On the whole, the greater number of authorities unite in finding that a small quantity is given off. But of late, much confidence has been reposed in the experiments of Dr. Edwards, who found that azote is sometimes absorbed, and sometimes given off, according to the season of the year. M. Collard de Martigny is at variance, however, with Dr. Edwards on this point, having invariably found in many trials at different seasons that a small quantity of azote is given off. In the experiments formerly mentioned to determine the proportion of oxygen which disappears in respiration, he found in four trials with 244 cubic inches of air, that azote was exhaled to the amount of 1.9, 1.8, 1.6, and 0.1, cubic inch; in four trials with 213 cubic inches, the quntity exhaled was 4.1, 3.6, 1.8, and 1.3 cubic inches; and in one trial with 183 cubic inches, the quantity was 4 cubic inches.

5. Lastly, M. Collard de Martigany considers the question, whether any water is formed in the lungs by the union of the oxygen of the air with the hydrogen of the blood. The idea that water is so formed has been, we believe, universally abandoned in Britain for some time, and we were not aware that this branch of the Lavoisierian doctrine of respiration still met with its favourers in France. It may be at the same time true, as our author states, that it is a notion more easily rejected than disproved. The objection first urged against it, that hydrogen never unites with oxygen at so low a temperature as 100 degrees, was met with the rejoinder, that such union readily occurs when the hydrogen is in a *nascent* state. But M. Collard de Martigny objects, that according to his own experiments, and those of M. Chevallier, hydrogen is never in a nascent state brought in contact with azote without amonia being formed, which he has never found in the halitus of the respiration. Another objection is, that whether common air or azote be respired, the quantity of halitus formed is pretty nearly the same.

In a paper which will appear presently, the author of the present essay undertakes to prove that *animal heat is altogether independent of respiration.*

2. *Observations on the influence of imperfect supplies of fresh air, long continued, on the general health.*.—By William Watson, Esq., Surgeon, Wanlockhead.—From the experiments of Lavoisier, Davy, Allen and Pepys, and Dr. W. F. Edwards. we learn, that air, containing a certain proportion of oxygen gas is indispensable to effect the requisite changes on the blood of the pulmonary artery; that in the course of successive respirations, the oxygen gradually diminishes, and that its place is supplied by variable proportions of carbonic acid gas; and that, in consequence of these changes in the air respired, frequent fresh supplies are requisite. It was also ascertained by Allen and Pepys, that when an animal breathes the same quantity of air without change, death ensues before all the oxygen disappears; and that when the same portion of air is respired until it can no longer effect the requisite changes, it still contains only 10 per cent. of carbonic acid.

In all the experiments of these observers, the animals were made to respire an atmosphere which was gradually becoming vitiated, until insensibility or fatal *asphyxia* took place; and no attention was paid to the question, how far an atmosphere not frequently changed is capable of supporting life, and what influence such an atmosphere exercises on the functions and health of the animal body?

This question, I think, may be illustrated, if not determined, by the situation of miners, who are under the necessity of following their avocations in places so remote from the general mass of the atmosphere as to be almost insulated; and where, of course, there is neither a regular ingress of atmospheric air, nor egress of the substance formed during respiration and combustion. In these places the air is so rapidly inhaled, that a considerable diminution of the quantity of oxygen must follow, and its place being supplied by an equal quantity of carbonic acid gas, the bulk remains unaltered. The carbonic acid, however, from its specific gravity, falls to the bottom of the mine, where its presence is indicated by a candle burning more obscurely than when placed at a greater height.

But carbonic acid is scarcely ever accumulated in such quantities as to effect the action of breathing, except in so far as that is connected with the process of oxidation and assimilation, and then its effects are very material; since all the nutritive particles, before they can be assimilated with the blood, must be submitted to the action of the atmosphere, through the medium of the lungs. "In this way," to use the words of one of our ablest philosophers, "respiration may be said to be carried into every part of the body." Since, then, a certain proportion of oxygen is so necessary for the due support of the animal system, and since it has been demonstrated by satisfactory experiments, that a medium, consisting entirely of oxygen, would excite the action of all the vital functions to such a degree as even to cause the extinction of life, it seems a natural inference, that the inhalation of a medium, deprived of a part of its oxygen, should be attended with very different results—namely, a loss of tone, or debility in proportion to the quantity inhaled.

That this is really the case, is not only proved by the experiments above alluded to, but all the observations which I have made during a considerable number of years tend to strengthen my conviction of its truth.

In the year 1823, sixteen men were employed, during four months, in a mine where the deficiency of air was so considerable, that a candle would not burn for any length of time, except when the wick was made so open as to allow the greatest possible quantity of air to come in contact with the ignited part. The candle was placed generally in a sloping position. Notwithstanding the deficiency of air, none of the miners had any inflammatory complaints, but all complained of lassitude, debility, and drowsiness, particularly towards the end of each day's work, sixteen hours; and they all became gradually paler in complexion.

In 1824, a number of men were employed in the same mine, with nearly the same result. Thirteen were quite free from any particular complaint,—one complained, for a few weeks, of a slight pain in the stomach,—the other two, for a few days, of pain in different parts of the body from obstructed perspiration.

In 1826, sixteen men were employed five months in a mine where the quantity of air was still smaller than in the former—still the miners were free from any particular complaint. Only one complained of shifting pain, from improper exposure when perspiring freely. In 1827, the same number of men were employed in the same mine for four months, and all remained free from complaint. Reasoning, then, from analogy, it seems fair to conclude, that a person placed in a medium where there is considerable deficiency of oxygen, must become less liable to inflammatory action, and that this non-liability will be in direct ratio to the length of time he is confined in such medium. At least, this is no speculation founded on mere conjecture, but supported, in my opinion, by the facts which have come under my own observation. From these, I draw the following inferences:— 1st, That miners are not more liable to inflammatory complaints than any other class of the community. 2dly, That a majority of them, at least, in this district, remain free from any particular affection of the chest. 3dly, That hydrothorax among miners, is rather a rare disease, only two cases having occurred to me in the course of fifteen year's practice. With regard to the first of these conclusions, I may merely mention, that a majority of the inflammatory affections which have come under my notice, arose from exposure to sudden vicissitudes of weather, as in the case of engine-keepers, lead-washers, carters, &c.; and as to the second,—of sixteen miners, each sixty years of age or upwards, and still connected with the work, four have been invalided; while the other twelve continue to follow their occupations as usual. The united ages of the miners amount to 329.

I may also observe, that from a professional observation of fifteen years in this mining village, I have been led to the conclusion, that phthisis is not more prevalent among the miners here, than among any other description of people who are constantly inhaling pure atmospheric air.

As a proof of this, during the above-mentioned period, cases have occurred as frequently among females as among males; and even of the latter, there were several individuals who had never been employed in the mines at all—so that, with reference to this disease, the balance seems to be in favor of mining as an occupation; the deaths having been, according to my computation, in the proportion of six miners to nine others. I infer from this, also, that the effect generally attributed to the inhalation of noxious vapor and fine mechanical particles, namely, the irritation of the mucous membrane of the bronchia, and the inflammation thereby induced, has been much overrated; and that the conclusions drawn from this supposed effect are founded rather on analogy than on experiment or observation. The idea that fine mechanical particles may be suspended in the atmosphere, and, being inhaled along with that fluid, may produce the irritation alleged, I am inclined to receive with considerable distrust. Mechanical particles, to occasion any serious injury, must be of such a magnitude as would preclude the possibility of their being retained in suspension; and, on the other hand, such as from their extreme minuteness would admit of being so suspended, may either be inhaled with impunity, or must do their mischief in some other mode than the one assigned. It appears to me, therefore, that if consumption does occur more frequently among miners than among some other classes of the community—a position which I think questionable—it must arise chiefly from the laborious exercise necessary in their daily avocations, which, by accelerating the current of the blood through the lungs, may occasion some injury to that delicate organ, especially if any part of the vascular system was predisposed to disease. But to this I would consider them no more liable than any description of laborers on the surface whose stated exercise is equally severe. I am aware that this view of the subject differs from that of many medical gentlemen of established name—yet it has not been hastily or carelessly adopted, but is founded on long observation and experience; and, unless it can be invalidated by opposite experience, I flatter myself that the liberal and enlightened practitioner will hesitate to pronounce it altogether erroneous.—*Ed. Med. and Surg. Jour.*

3. *Phlebitis of the Saphena Veins cured by Compression.—May* 27, 1830.—A young man, æt. seventeen, was admitted into the Hotel Dieu, who had been obliged to remain constantly in a standing posture. He was of a feeble habit, and complained of pain and debility of the lower extremities. He had had diarrhœa for two days. This symptom soon disappeared, but the pains in the legs increased. Baths were employed without advantage. The continuance of the pain led to a more attentive investigaion of the case; and on the 7th of June, upon examining the lower extremities, small and irregular enlargements, of a bluish color, were detected upon the course of both internal saphena veins. These enlargements were very painful on pressure, and did not disappear under the weight of the finger. The inguinal glands were not tumefied. There were no red streaks of the skin, indicative of any affection of the lymphatic system. The patient could not bend his knees without pain, on account of a slight swelling in the ham, which was supposed to depend upon enlargement of the glands of the part, from obstruction to the venous circulation. The pain did not run in the track of the nerves, and was not, indeed present, except when the swelling was pressed upon, when it became very acute. There was little or no fever.

The case was now considered to be partial phlebitis of both saphenæ veins, and M. Recamier prescribed free doses of tartar emetic, which caused vomiting. No benefit was derived from this treatment, which, however, was scarcely continued long enough to enable any opinion to be formed of its efficacy. Compression was determined upon. Firm poultices of linseed were applied, and bound down by rather tight bandages.

The following day, the tumors on the veins were diminished in size, and less pain was felt on pressure. There was an obvious amendment every day, and in a short time all traces of phlebitis had ceased. Compression was still, however, continued, as the pains in the legs had not entirely disappeared.

It is to be observed, that the pains continued some time after the tumors, which showed the character of the disease had diminished. The limbs at length recovered their strength and action, and the patient quitted the hospital entirely cured.—*Journ. Complementaire.*

4. *Abdominal Pressure in Obstetrics.*—The custom of applying a bandage or swathe to a female *after* accouchment, is general, if not universal. It is not, however, so commonly known as it ought to be, that the same application, vigorously made, is a great aid to the expulsive efforts of the uterus. Pressure by the hand of a friend is almost always inefficiently applied; and much time might be saved the pratitioner, and much suffering the patient, if nurses were taught to press with sufficient force, and in the right direction, and at the proper times, on the uterus, during the pains of parturition. The following statement from the London Review, contains a practical lesson on this mode of procedure.

Mr. M'Morris, a medical officer in the East India Company's service, writes to us as follows. "In one of the numbers of the Medico-Chirurgical Review for 1828, I find a bandage proposed by Mr. Gaitskell for obstetric purposes. I have no doubt it will facilitate the parturient powers of Nature by the support and pressure which it affords. It is a common auxilliary among the native women on this side of the Indian peninsula, when the progress of labor is slow. Some of the female attendants, in such cases, place their hands on the abdomen of the parturient patient, and press it pretty violently downwards towards the pubes, having first rubbed it with castor oil. This operation is continued till the child is expelled, which generally happens soon after the mechanical pressure is commenced. If the secundines are not expelled with the fœtus, the woman is left to rest, with a bandage applied pretty tightly round the abdomen. I have been astonished in some cases that I have witnessed, to observe the increase of the expulsive pains soon after this process is put in force."

5. *Remarks on the Operation for the Hare-Lip.*—By Zadoc Howe, M. D., of Billerica, Mass.—Having observed that the operation for the hare-lip generally leaves more or less of the original deformity, I several years ago, adopted a mode of operating a little different from that described by surgical writers upon the subject.

The improvement consists in making two curved incisions, instead of two straight ones, in such manner that the opening, when ready to be brought together, shall resemble half of a leaf of the appletree, cut traversely, rather than the letter V, as directed by authors.

I first used this kind of incision in a cancerous affection of the under lip. The disease lay on the lower part of the lip, and a little down upon the chin; it was thought expedient to cut through the lip, for the sake of uniting the parts by the first intention: the curved incision was used at the time rather with a view to save the destruction of sound parts, than any improvement in other cases. I was gratified with the appearance of the lip when healed, and have always adopted this mode of operating since, in the hare-lip, whenever circumstances would permit.

After the operation, the edge of the lip, instead of drawing in, will turn a little out, giving it more of a natural appearance; and as the perpendicular cicatrix will be somewhat lengthened, no notch will appear in the edge; a circumstance which is very liable to happen after this operation, performed in the usual way. In short, it leaves rather less deformity; which all will allow is an object of some importance in operations upon the face. I suggested the substance of these remarks to the late Professor Smith, of New-Haven, several years ago. He procured a small pair of curved forceps, with a screw in the handles, on the principle of those used for breaking a stone in the bladder. His intention was to fasten the instrument upon the lip, and cut by the side of the blades. How the doctor succeeded with his instrument I never heard.

I have always cut by marks made with ink; and, in the hare-lip, always carry one interrupted suture, with a small needle, as high as possible in the nostril, that no fissure may appear when the head is elevated. One small gold pin, a little curved, together with adhesive straps, completes the dressing. I frequently give the incision a greater degree of curvature upon one side than the other, for the sake of saving the parts—always taking care to make the sides exactly correspond in length, and withdraw the pin in about sixty hours.*—*Am. Journ. Med. Sc.*

6. *Effects of Mercury—Taliacotian Operation.*—A case is recorded in a late periodical, in which extensive exfoliations of the bones of the palate and nose succeeded the free use of mercury. The pa-

* The preceding ingenious method of operating for hare-lip, lately suggested itself to Dr. J. R. Barton, of Philadelphia, and he actually put it in practice, in September last, in the case of a boy in the Pennsylvania Hospital. Dr. Barton supposed the method to be original with himself.—*Ed. Bost. Med. and Surg. Jour.*

tient had never had syphilis.—In this and two other cases, the columna nasi was restored, by Mr. Liston, by a strip of the upper lip, cut quite through and turned up. In his account of the operation, Mr. L. observes that the lateral slip, destined to form the new columna, should not be twisted round, so as to present its cutaneous surface externally, but merely elevated and affixed to the point of the nose. By twisting, the chance of success is diminished, and if the part does adhere, it is thick and clumsy. The mucous membrane forming the inferior surface of the columna, retains some of its characters for a few weeks, but gradually assumes a cuticular appearance. For some time after the operation, on tickling or compressing it, the sensation is referred to the inside of the mouth. Though it might be supposed that, in a male adult, the beard on the inner surface of the new columna would prove a source of irritation, this is not the case; the hairs lose their stiff and bristly character, not being cropped frequently as before, and being constantly moistened by the mucous secretion of the nostrils. In fact they come to resemble those hairs that grow naturally from the parts.

In the above cases, the incisions always adhered by the first intention; and I should think that if proper care be taken to place and preserve the raw surfaces in accurate contact, adhesions will always occur, little or no mark remaining. Indeed, the appearance of the upper lip will always be benefitted by the operation, for when the columna is lost, the lip becomes elongated and tumid at its centre; and by a narrow slip being removed from it, this defect is obviated, whilst the cicatrix, from being in the situation of the natural depression, is scarcely observable.

In the after-treatment, it is necessary to keep the alæ tense by dossils of lint, in order to assist the columna, not yet sufficiently consolidated, in supporting the parts in their natural situation; and when the columna itself becomes tumid, a compress and bandage should be neatly applied over it.

When the cartilaginous septum is destroyed, as is generally the case, of course an aperture remains between the new columna and the oseous septum, but that is not perceived unless on close examination, and does not annoy the patient.

In a recent number of the Journal Hebdomadaire, a case is recorded, in which attempts had been made to restore a lost columna, first by M. Dupuytren, of Paris, and afterwards by M. Gensoul, of Lyons. The operation failed, and the nose became approximated to the lip. M. Dupuytren raised a *flag of integument* from the lip, and adapted it to the nose, after having *twisted it round.* Had he formed his columna from the *whole thickness of the lip,* there would have been no necessity for *twisting* it, and in all probability the operation would have proved successful.

7. *Acetate of Morphia in various Diseases.*—Dr. Bardsley, in his "Hospital Facts and Observations, &c.," a work already quoted in this Journal, has entered somewhat largely on the use of the acetate of morphia in various diseases. He has found it particularly useful in cases of pain at the pit of the stomach, in pyrosis, in uterine diseases, and in neuralgia. Not having the work before us at this moment, we shall quote a few cases, as abridged in the Medico Chirurgical Review for October last.

Michael Sharples, aged twenty-nine, an industrious man with a numerous family, had experienced much pain at the stomach occasionally for several months; and so severe had it been for the last three weeks, that he could not follow his usual employment. His spirits were low, appetite gone, and bowels costive. Leeches and a blister had been tried. The bowels being open by purging medicine, a quarter of a grain of the acetate was given three times, daily, with excellent effect. In five weeks he was discharged cured, but he subsequently applied with a relapse, which was combatted successfully by the same plan of treatment.

We will cite another case.

A young man had suffered, for some months, from such a gnawing pain at the stomach, that he could not continue his occupation, was reduced to poverty, and at last attempted to poison himself with laudanum. After a short course of aperients, Dr. Bardsley gave half a grain of the acetate twice, daily: so much relief ensued that the dose was diminished; and after the expiration of several weeks, he perfectly recovered both his mental and bodily strength.

In the only cases of pyrosis which Dr. Bardsley details, the patient, a female, aged twenty-six, had always been delicate but suffered severely, for the last two months, from gnawing pain at the pit of the stomach, copious discharge of an acid fluid from the mouth, flatulence, and capricious appetite. After castor oil, the acetate was prescribed—a fourth of a grain twice daily; and in six weeks the patient was discharged cured.

It is not to be supposed, however, that Dr. B. regards the acetate of morphia as a sovereign remedy in all cases of dyspepsia. So far from this, he alludes to several cases of the disease in which the remedy was productive of little or no relief. This is exactly what we should have anticipated, from the known effects of the remedy, and the diversity that exists in the nature and characters of dyspepsia. Dr. B. does not appear to think as favorably of *opium* in stomach complaints as the morphia; and among the cases he details, we notice some in which the former of these remedies had been used ineffectually, before Dr. B. resorted to the latter. In our own practice, we have seen opium very useful, and even completely scccessful in that complaint. In all such cases, the pain recurred after eating, and continued so long as the articles ingested remained in the stomach. Under such circumstances, a small dose of opium, given half an hour before meals, and repeated during a week or two, sufficed to effect a cure. It is very plain that in these cases the pain was nervous, and not the result of inflammation, and that care was taken to ascertain the true nature of the disease before resorting to this plan of treatment.

Dr. B. has used the same remedy, with palliative and remedial effect, in scirrhus and induration of the uterus, and particularly in painful menstruation. In a case of the latter disease, he ordered the patient "to take a quarter of a grain of the acetate of morphia on the first accession of pain, and to continue this proportion every half hour, whilst it was violent. The second dose afforded her very great relief, without in the least suppressing the discharge;" and at the time Dr. B. was writing, she took the morphia at every monthly period, with the same advantage.

Nor did Dr. Bardsley use the morphia with less advantage or success in neuralgia of the face, forehead, lower jaw, &c. In all these cases, the remedy was administered internally. On the subject of this remedy, Dr. B. makes the following general remarks:—

"The above examples seem sufficient to establish the remedial efficacy of the acetate of morphia in several affections. It must be allowed that morphia has not always answered the intentions with which it had been employed; but the proportion of the favorable cases has been considerable, compared to those in which it has failed. I have never witnessed any *pernicious consequences* from a prudent use of the morphia. I am led to recommend the acetate of morphia in preference to opium, from a conviction that its efficacy may be equally relied upon, whilst its administration will be unattended by the *distressing headach, excessive constipation, and other unpleasant symptoms*, which that drug in large doses, mostly induces. It appears to be the chief advantage of morphia, that it may be employed in those cases in which it is desirable to obtain a narcotic effect, and at the same time, of the first importance to avoid constipation. It is proper to unload the bowels before commencing with the acetate, so as to give the remedy a fair chance. It is prudent to commence with not more than a quarter of a grain, which may be gradually increased to half a grain, a grain, or two grains, according to the urgency of the symptoms and the effects produced. These doses are applicable to adults. I have mostly prescribed it in this form of pill, which seems to answer very well."—*N. A. Med. and Surg. Journ.*

8. *Cause of Stammering.*—In our sixth volume, p. 233, et seq. we published Dr. Arnott's explanation of the nature of stammering; and we find in a recent No. of the *Journal of the Royal Institution of Great Britain* some interesting observations on this explanation, by Marshall Hall, M. D. which we will now lay before our readers.

Dr. Hall is of opinion that Dr. Arnott's view of the subject is so far from being correct, that it is quite plain that it is only in the articulation of certain letters that expiration is interrupted, and even in this case the interruption is not in the larynx, the organ of voice, but in some part of the mouth, or organ of speech. "It will assist us," says Dr. Hall, "in the determination of the question, to take a review of the influence which the natural articulation has upon respiration, or rather upon expiration. It may be ascertained, by the simplest experiment, that in the pronunciation of the short word BAT, we adopt a mechanism, by which not only the different letters are formed, but the respiration is twice completely arrested;—and that, in the pronunciation of the equally short word FAN, we first interrupt the flow of the air through the nostrils whilst it is forced between the teeth and lower lip, and then intercept the course of the air through the mouth, whilst we allow it to pass only through the nostrils.

"It is on their influence on the respiration, that I formed the division and arrangement of the consonants, published in the nineteenth volume of this Journal; their sub-division was founded on the respective mode or mechanism of their enunciation. I divided them—

"1. Into those, in the articulation of which both the mouth and the nostrils are closed, and the respiration, of course, completely arrested.

"2. Into those, in the enunciation of which the nostrils are closed, but the mouth left more or less open, for the exit of the air, which is compressed, but not interrupted in its expiration.

"3. Into those, not requiring even the nostrils to be closed, and in the enunciation of which the air is still less compressed in its course from the lungs: and,

"4. Into those, in the articulation of which the expired air is not interrupted, and scarcely impelled at all.

"Of the *first* class, are

B D G*
P; T; K.

"In tracing these letters into their sub-divisions, we may observe, that the first pair are labials, being formed by the lips compressed together; the second pair are linguo-dentals, formed by pressing the point of the tongue against the posterior and upper part of the upper teeth; and the third pair are linguo-palatal, being effected by pressing the middle part of the tongue against the palate. In all, the posterior apertures of the nostrils are effectually closed by the pendulous vail of the palate being drawn upwards, and accurately applied to their posterior apertures. And of course, those persons whose palate is perforated, or in whom the pendulous vail of the palate is imperfect, as sometimes arises from disease, are more or less incapacitated from pronouncing these letters, the expired air being no longer intercepted, as it ought to be, in its course.

"Of the *second* class, are

F/V; the TH†; and S/Z.

"In the articulation of these letters, the posterior orifices of the nostrils are required to be closed, whilst, in the first pair, the compressed air is continually forced between the upper teeth and the under lip; in the second, between the teeth and the tongue; and in the third, between the point of the tongue and the anterior part of the palate.

"From this view of the subject, it will be readily apprehended how the substitution of D or T for the TH, by foreigners, is so remarkable;

* i. e. the *hard* G. † *Hard* and *soft*.

for it is no less than the substitution of a total interruption, for a mere compression of the air, in its exit from the chest.

"Of the *third* class of letters, are

M; N; L; R.

"In the enunciation of these letters. the expired air is only very slightly compressed, the nostrils being left freely open. It is for this very reason, probably, that these letters have been termed *liquids*, as flowing without obstacle. And it is by this circumstance, principally, extraordinary as it may appear, that the letter M differs from the letters B and P, for they are all equally labial; and that the letter N differs from T and D, for they are all equally formed by placing the point of the tongue near the roots of the upper teeth.

"Of the *fourth* and last class, are

H; the Greek X; Y; and W.

"In the enunciation of these consonants, the air appears to be scarcely compressed or impeded in its exit at all. This fact may, I think, account for the circumstance, that it has even been doubted, whether the two last letters be really consonants or not; and for the remarkable fact, that they cannot, as *consonants*, form the termination of any word. Their mechanism is guttural, double dental, andlabial, respectively.

"These letters, preceded as they are in this arrangement, by the liquids, lead us almost insensibly to the class of letters to be next noticed, namely, the *vowels*.

"These are so called, from having been supposed to relate to the *voice* alone.* This, however, is obviously an error. The different parts forming the mouth, or organ of *speech*, are not less necessary to the enunciation of the vowels, than to that of the consonants, or their function less appreciable, on carefully making the experiment. Thus, the French U is entirely labial; the letter E is dental; O, palatal; whilst the dipthong AW, and the vowels marked in the French language by the circumflex, (ʌ,) are guttural.

"Now let any one carefully examine the effort made by the stammerer in his attempts at the enunciation of these various letters. It will be obvious that the malady is but an exaggeration of the natural effort. In attempting to pronounce the letters of the first class, violent efforts are made, yet expiration—articulation—is not effected; but there is frequently, nay generally, a peculiar noise heard in the larynx, although its full enunciation is prevented by the action of the muscles of the mouth. But if the letters of the second class are pronounced with stammering, there is a perpetual hissing from the escape of compressed air, in the case of the letters F and V, between the lips, in that of the TH, between the tongue and upper teeth, and in that of the letters S and Z, between the teeth. In the stammering enunciation of the letters of the third class, there is frequently a state of laborious respiration. In all these cases, then, it is plain that the larynx is open; any considerable effort applied to the parts concerned

*Blumenbachii Institutiones Physiologiæ, Ed. MDCCCX, Sectio IX.

in the articulation of the first classes of letters—the least noise—the least escape of air, alike demonstrate this fact. In the natural, and in the stammering articulation, there is the same total or partial interruption of the expiration, at the same parts, not of the larynx, but of the proper organs of articulation, only in different degrees. Let the larynx be really closed, which may be done after a little trial, and it will immediately discovered that stammering is, in fact, impossible; the effort made by the force of the expired air against the parts of the mouth called into action in the articulation of the first class of letters —all escape of air—all noise, become totally interrupted.

"I have just attentively watched the attempts of a stammerer to articulate the various letters.

"In the effort to pronounce the first class of letters, especially the letter T, still more if T's come together, as in the words THAT TREE, the face became flushed even from interrupted expiration; yet there was, at every repetitition of the effort, a noise audible in the larynx, proving that this part was unclosed.

"In pronouncing the letters of the second class, a repeated hissing noise was distinctly produced by the flow of the compressed air, in one case, (F, V,) between the under lip and upper teeth; in the second, (TH,) between the tongue and upper teeth; and in the third (S, Z,) between the teeth.

"In attempting the articulation of some of the letters of the third and fourth classes, and some of the vowels, the breath was sometimes lost, as it were, in a full and exhausting expiration, altogether peculiar.

"All these results prove that the larynx is not closed in stammering, and indeed that its closure and stammering are totally incompatible with each other. When expiration is interrupted, it is by the co-operotion, the co-adaptation, of parts anterior to the larynx; it is, in a word, not an interuption in the organ of voice, but in that of speech. The paralysis of the laryngeal muscles could not, therefore, effect the good which Dr. Arnott ingeniously supposes.

"But would no evil really result from this paralysis of the muscles of the larynx? Would the 'loss of the faculty of closing the larynx' really 'be of no moment?' On the contrary, the accurate closure of the larynx, not by the epiglottis, but by means of its own muscles, is essential to the act of deglutition. This is demonstratively proved by M. Majendie, in his interesting memoir, 'Sur l'usage de l'Epiglotte dans la Deglutition.' The fact is further proved by cases of actual paralysis of the laryngeal muscles occurring in the human body, and by the effects of inflammation and contraction, and of ulceration of the internal parts of the larynx, inducing defective deglutition."—*Amer. Journ. of Med. Sciences.*

9. *Itching of Delicate Surfaces.*—Dr. Ruan, a Parisian physician, of celebrity in a communication to Professor Hufeland, of Berlin, states, that he has succeeded in some distressing cases of incessant itching of parts of the body, covered with skin of very delicate structure, after opiates and internal and external remedies had totally failed, by the exhibition of the balsam of copavia in the dose of twenty-five drops three times a day. In two cases in which it failed, a solution of the borate of soda (a dram to half a pint of fresh elder-flower water), used as a lotion, was employed with success. In another case, a permanent cure was obtained by sprinkling the parts, which were excoriated by scratching, with a powder composed of starch and the levigated carbonate of zinc.

Dr. Dewees, in his treatise on the Complaints of Females, has noticed a few cases of this most distressing malady, in which balsam copavia and a lotion of the borate of soda speedily and perfectly succeeded.

This disease is probably a variety of chronic erysipelas, differing from that of the skin of the face, neck, and chest, from the peculiar delicate texture and secretion of the parts in which it occurs. It is a curious fact, that a topical application, which in one case has had a speedy effect in curing it, in another apparently exactly similar in every respect, will often aggravate the disease. Generally speaking, we have found the constant application of a solution of acetate of lead with acetate of morphine, in fresh elder-flower water, with attention to the stomach and bowels, and the internal use of the blue pill and extract of cicuta, to succeed, it some obstinate cases, the ointment of the prussiate of mercury has had a very happy effect, having succeeded in effectiag a cure in the course of a few days. After rubbing a little of this ointment over the surface, it should be applied, spread on fine lint, so as to prevent the surfaces coming in contact. In cases of long standing the cuticle is so much diseased, that no lotion or ointment can succeed till it has been destroyed by a weak solution of nitrate of silver, which, so far from increasing the irritation, considerably allays it. We have met with cases which were aggravated by oily or greasy applications and by lotions, which gave way to the frequent application of a mixture of plain hair-powder, and a small proportion of hydro-sublimed calomel.—*Gaz. of Pract. Med.*

10. ***Effects of Posture of the Pulse in Health and Disease.***—It is known that considerable variety, both in strength and frequency of pulse, is observed in the erect, the sedentary, and recumbent postures. Dr. Thompson, of Edinburgh, first pointed out some of these differences from an extensive series accurate observations; and these were confirmed by Dr. Stroud, in the numerous experiments made by him on the patients of the clinical wards in the years 1816 and 1817. Unaware of these experiments apparently, Dr. Graves has, with extreme diligence, devoted himself to the observation of the varieties of the pulse, not only in the erect and recumbent posture, but in the inverted, with the feet above and the head dependant. The author found, however, that this position produced less effect than he anticipated. The frequency of the pulse in this position, was neither retarded nor accellerated; but its strength was diminished, sometimes very considerably, and occasionally it became very irregular: a circumstance which Dr. G. is inclined to ascribe to the weight of the column of blood pressing on the aortic valves, and thus augmenting the impediment to its exit from the ventricle.

Dr. Graves found the pulse less frequent, from six to fifteen beats in the minute, and stronger, in the horizontal than in the erect posture, and he infers, that in this position its maximum of strength and minimum of frequency are at once attained. This fact he thinks may explain the relief obtained by placing patients in the horizontal position in order to avoid fainting, for instance, that produced by venesection. This, however, with deference to the author, we must be permitted to question. Syncope from venesection is doubtless occasioned from the blood being so suddenly drawn off, that there is no longer a sufficient quantity left to excite the action of the heart; and it is with equal certainty obviated by placing the patient in that position, in which, while the blood is drained off from the inferior vessels, it may, by being gradually allowed to flow from the superior ones, still be adequate to excite the heart.

In all diseases, except cases of hypertrophy with dilatation of the heart, Dr. Graves found the pulse to differ in frequency both in the erect, sitting, and horizontal postures. In four of these six cases, the fact of hypertrophy with dilation was ascertained by inspection; and of the other two, in one it was certain during life, and in the other was at least probable. It would be doubtless premature to speculate on the cause of this peculiarity, until the phenomenon has been observed in a greater number of cases. We conclude our account of this paper by subjoining the general conclusions, which Dr. G. thinks may be established from an extensive number of observations.

"1. That the greatest difference occurs in patients laboring under fever, or in a debilitated state, in consequence of fever or any other cause. It may amount to thirty, forty, or even fifty, between the horizontal and erect postures.

"2. That this difference decreases after the first quarter of an hour, in most cases, but always remains considerable as long as the same position is observed.

"3. That in persons not much debilitated, the difference is much less than that stated above, and often does not amount to more than ten.

"4. That when the patient lies down, the pulse rapidly falls to its former standard.

"5. That in some the frequency is greater between the horizontal and sitting posture, than between the latter and the erect, while in others the contrary takes place, so that generally the frequency of the sitting posture may be taken as a mean.

"In persons convalescent from fever or acute diseases, I find it is extremely useful to the physician to ascertain the comparative frequency of the pulse in the horizontal and erect position. The greater the difference, the greater is the debility of the patient; and, consequently, the more guarded must his medical attendant be in allowing him to sit for any length of time, particularly if the pulse, on his lying down, does not resume its usual degree of frequency."—*Edinburgh Med. and Surg. Journ.*

11. *On the Compound Syrup and Fluid Extract of Sarsaparilla.*—Since the publication of Dr. Hancock's paper in the Transactions of the Medico-Botanical Society of London, and which was copied into the Journal of the Philadelphia College of Pharmacy (vol. i., p. 295), there can remain but little doubt that the frequent inefficacy of the sarsaparilla arises chiefly from the careless or the erroneous manner in which it is generally prepared. Of all the forms in which this important remedy has been exhibited, perhaps none has obtained so little justice in its preparation, and consequently in the estimation of those who have to judge from its remedial effects, as the syrup. It is now generally conceded, by those who have paid any attention to the subject, that the effective principle of sarsaparilla like that of many other vegetables, is entirely destroyed by long exposure to a boiling heat; yet all our authorised formulæ for the preparation of the syrup are liable to this objection. As far as my knowledge extends, they all direct it to be made by decoction in water, or by long-continued hot infusion; and we have invariably, in consequence, two evils to endure—the presence of a large quantity of feculent and mucilaginous matter, which causes rapid decomposition; and, what is still worse, the absence of nearly all the active principle of the root, which has indeed been effectually *stewed down.* The French Codex, for example, directs two pounds of the roots to be infused for twenty-four hours in twelve pounds of warm water, then boiled a quarter of an hour, and the residuum submitted to another, and still another boiling, with ten pounds more water each time down to six. The mixed decoctions are then to be again boiled, with the senna, &c., down to one-half; after straining, the sugar and honey are to be added, and the whole boiled a fifth time, in order to form the syrup! Swediaur, in his *Pharmacopœia Medici Practici Universalis*, orders to boil the sarsaparilla twice in successive portions down to one third (nine pints down to three), and repeated; then to infuse the senna, &c.; and afterwards to boil again with the sugar and honey. The Antwerp *Pharmacopœia Manualis* directs the root to de infused in hot water for twenty-four hours, the infusion stewed down to one third; this operation repeated twice with the dregs; the mixed liquors then reduced to one half; the herbs infused towards the end of the evaporation; and lastly, the sugar and honey to be boiled to a syrup in the infusion. It would be tedious to particularize further the various formulæ for this preparation, which are more or less similar to those I have detailed. The American Pharmacopœia of 1820, differs in no essential point. The London College has no compound syrup, but directs the simple to be made by maceration of the root in boiling water for twenty-four hours, evaporating to one half (which must be done by continued boiling), and, lastly, boiling and evaporation again after the addition of the sugar, to form a syrup of proper consistence. The boiling point of such a compound being higher than that of water, subjects it to an obvious additional disadvantage. Indeed, by a strange mistake it seems to have been generally taken for granted, that long boiling was the best, instead of the worst mode of extracting the active principle from the sarsaparilla. Can we therefore be at a loss to account for the fact, that our *sirop de cuisinier* has none of the sensible properties of the sarsaparilla, and often requires the assistance of mercury to give it any effect whatever?

To obviate the disadvantage of so inert a preparation, I determined, some months ago, to try a different process, and I have obtained a syrup, which has in perfection the characteristic acridity of genuine fresh sarsaparilla, and discovers no tendency to fermentation through the hottest part of our summer. The following is the formula which I can recommend for general adoption.

R. Rad. sarsaparillæ contus. lb. ij.
Rad. glycyrrhizæ contus.,
Fol. rosæ rubræ,
Fol. sennæ aa oz. ij.
Ligni guaiac. rasi, oz. iij.
Spir. vini tenuioris. O. x.

Digest for fourteen days at a common temperature; then strain, express, and filter, evaporate the tincture by a water bath to four and a half pints (so as to get rid of the alcohol), then add, white sugar, lb. viij., and form a syrup, removing it from the fire as soon as the sugar is dissolved. When cold, add

Ol. anisi, gtt. vj.
Ol. gaultheriæ, gtt. iij.
Ol. sassafras, gtt. vj., previously rubbed down gradually with a little of the syrup. From these quantities I obtained seven pints of syrup, possessing, as I have said, the double advantage of the perfect extraction and preservation of the active principle, and the absence of any fermentable matter.

A somewhat analogous preparation of the sarsaparilla has lately been introduced into practice, under the name of the Compound Fluid Extract, which, if properly prepared, would contain the sarsaparilla in a concentrated state, but which has hitherto suffered the same fate as its milder relative, the syrup. It has generally been prepared by evaporating the compound decoction and adding a little sugar; and consequently shares the disadvantages of that preparation in a still greater degree.* Applying to this preparation the principles on which I made the syrup, I have obtained a compound fluid extract of a very superior quality, and possessing in an eminent degree the active properties of sarsaparilla. The following is the process:—

R. Rad. sarsaparillæ contus. oz. xvj.
Rad. glycyrrhizæ contus.,

Ligni guaiac. rasi,
Cort. rad. sassafras aa oz. ij.
Cort. rad. mezerei, oz. vj.
Spir. vini tenuioris O. viij.

Digest for fourteen days, at a common temperature; then strain, express, and filter. Evaporate the tincture in a water bath to 12 fluid ounces; then add of white sugar oz. viij., and remove from the fire as soon as the sugar is dissolved. Where the quantity made is considerable, it may be worth while to preserve the alcohol for future operations, by distilling instead of evaporating it. During the process a small quantity of resin separates by adhesion to the sides of the vessel, and which appears to be merely from the wood of the guaiacum. We have here a preparation, a fluid drachm of which contains in perfection the active principle of a drachm of sarsaparilla, and of which two fluid ounces are equal in strength, and similar in composition, to what one pint of the compound decoction of the Pharmacopœia would be, were it possible to prepare it without more or less destroying the active principle of the sarsa.—*Philad. Journ. of Pharmacy.*

12. *Case of Putrefactive Disorganization of the Lungs.* By Robert Law, A. M., M. B.—John Dunne, tailor, aged nineteen, of a thin delicate habit of body, was admitted into Sir Patrick Dunn's Hospital for fever, in the progress of which he was seized with a profuse expectoration of fluid arterial blood, which he said he had often had before his admission; his pulse was small and rapid. (R. Misturæ Camphoræ, v. drams; Tinctur digitalis, gutts. xxx.; Tinctur opii, gutts. xx.; Syrupi, ss. drams, Misce sumat unciam omni trihorio.) The hæmoptysis ceased; in the course of the fever, he exhibited symptoms which gave strong grounds for suspecting effusion in the head, by becoming comatose, the pupils widely dilated did not obey the stimulus of light; these symptoms, however, yielded to the application of cold wash to the shaved head, a blister to the nape of the neck, and sinapisms to the feet; his febrile symptoms soon disappeared, but he complained of a teasing cough and palpitation of the heart, for which I repeated the camphor mixture and digitalis, with the following pills:

R. Extract conii gr. viij.

Pilulæ ipecac. gr. iv. fiant pilulæ quatuor una tertiis horis sumend.

These lessened the heart's action and the irritation of the cough, but he was much debilitated, and perspired much at night. I in consequence ordered bark and sulphuric acid, and the tepid shower bath, from which he seemed to gain strength. I now lost sight of him for some time, when he came to me to complain of the unceasing irritation of the cough; he was much emaciated and decidely hectic; his breath and expectoration were extremely fœtid; in all the right lung respiration could scarcely be heard; under the left clavicle was imperfect pectoriloquy; he died in two days from this, about six weeks since his first admission into the hospital.

Examination fifteen hours after death; head not examined; the apex of the right lung adhered so firmly to the corresponding part of the cavity of the chest, that it could not be separated without breaking its structure; this lung was much heavier than natural, and felt quite solid, except at its base; the investing pleura was universally thickened, and in some places had acquired the density of fibro cartilage. The entire substance of the lung, except the base, was thickly studded with tubercles; the pulmonary tissue surrounding these bodies was either broken down into a soft brownish sloughy substance, or so condensed as to have its cellular nature quite destroyed; there were many irregular cavities traversed by bands of pulmonary structure; the surface of each cavity exhibited a blackish sloughy appearance; the base of the lung was quite free from tubercles, but was in the first stage of pneumonia.

The left lung exhibited a similar disorganized condition, the small irregular cavities were more numerous, and the intervening pulmonary structure softer, presenting the same dirty, sloughy, broken down appearance. On pursuing some of the bronchial ramifications which opened into these cavities, their lining membrane was highly vascular, and, in some instances, black; the base of this lung too, was in the first stage of pneumonia, and free from tubercles; the left cavity of the pleura contained about a pint of straw-colored serum; the pericardium, about eight ounces of the same fluid. There was also an infiltration in the sub-serous tissue, connecting the substance of the heart and the pericardium; the heart was small and flabby; the abdomen contained about two quarts of serum, all the viscera of this cavity were healthy.

We here have an instance of the disease, combined with tubercular phthisis, and, in consequencec, running a much more rapid course, and exhibiting a more distinctly marked hectic fever, than the uncomplicated disease ordinarily does. As usual, it was ushered in by a profuse hemorrhage, to which succeeded cough and fœtid expectoration; as in ordinary phthisis, the superior portion of the lung was the point de depart of the disease.

A striking circumstance in this case is, the effusion into all the serous cavities, which I suspect also took place into the brain, when the coma, dilated pupils, &c., gave evidence of its existence.—*Transactions of the Association of Fellows and Licentiates of the King and Queen's College of Physicians in Ireland*, *N. S. Vol. I.*

13. *Gaseous Tumors of the Uterus.*—Two very interesting cases of this description are related in the fourth volume of the *Opuscoli della Societa Medico-Chirurgica di Bologna.* As we have not received that work. we translate from the December No. of the *Revue Medicale* the following notice of them.

Case I. A plethoric female, in the prime of life, being suddenly exposed to cold during menstruation, the discharge was suppressed. Acute pain in the region of the uterus was quickly induced, as well as a marked augmentation in the size of this organ, which reached to the umbilicus. The patient was unable to move her lower limbs, or even her body. To the chill with which the disease was ushered in, succeeded fever with excerbations; in the evening there was thirst, and great uneasiness. On examination of the uterus, its tympanic condition was easily ascertained. A large bleeding from the foot, emollient fomentations, enemata of chamomile and of elder, did not at all diminish the condition of the uterus, which even became rounder: leeches were applied to the vulva with no more success. The symptoms constantly augmenting, another examination was instituted; the finger was introduced into the meatus uterinus, which gave issue to a quantity of fœtid gas. The size of the hypogastrium diminished very sensibly, but soon again increased. Fumigations by means of a tube introduced into the uterus were then resorted to, which caused a very copious discharge of gas, accompanied with clots of blood, which continued several days, and produced a complete cure.

Case II. *Gaseous Tumour of the Uterus simulating Pregnancy.*—A woman, aged forty, who had never had any children, exhibited some signs of pregnancy. The menses, which had always previously been regular, were suppressed; the meatus uterinus was entirely closed; the patient did not however experience any of the disorders which attend pregnancy. The uterus, however, towards the fifth month was as high as the umbilicus, moreover its form could be distinguished by pressure with the hand. Such was the condition of this woman, when towards the sixth month all these prospects vanished; in stooping, a great discbarge of flatus took place, the abdomen fell, and in a few days returned to its natural condition.

16 *Small-pox Infection Communicated to the Fœtus in Ultero without the Mother feeling any indisposition from its Action on her own Constitution.*—We published in our last No. p. 555, a case in which this very curious phenomenon occurred. Since the publication of that No. we have met with accounts of several cases in which a similar occurrence took place, so that it appears not to be quite so rare an event as our correspondent supposes. Dr. Edward Jenner relates in the first volume of the *Medico-Chirurgical Transactions* of London, two cases of this kind, and alludes to one mentioned by Dr. Mead, and he says that he is acquainted with more examples of a similar description. As this is so interesting a pathological phenomenon, and as the work in which these cases are published is rarely to be met with in this country, we shall give a brief account of them.

The first case is related by Dr. Jenner. Dr. J. was requested by Dr. Croft to vaccinate an infant, and scarcely any effect being produced beyond a little efflorescence on the part, which in a few days disappeared, Dr. J. expressed his surprise when the mother related to him the following particulars:

"A few days previous to her confinement, she met a very disgusting object, whose face was covered with the small-pox. The smell and appearance of the poor creature affected her much at the time; and though she mentioned the circumstance on her return home, she had no idea that her infant could suffer from it, having had the small-pox herself when a child. During a few days after its birth, the little one seemed quite well, but on the fifth day it became indisposed, and on the seventh the small-pox appeared. The pustules, which were few in number matured completely. Dr. Croft, who attended her, being curious to know the effect of inocculation from one of the pustules, put some of the matter taken from one of them, into the hands of a gentleman eminently versed in that practice, which produced the disease correctly. Mrs. W. was not sensible of any indisposition herself from this exposure, nor had she any appearance of the small-pox.

The following case, similar in its general character, was cummunicated to Dr. J. by Mr. Henry Gervis. During the prevalence of a small-pox epidemic, Mr. Gervis vaccinated a woman in the last month of her pregnancy. " Her three children had been inocculated the proceding day with variolous matter by the surgeon who attended the poor of the parish, and who had very properly declined inoculating her also, from her particular situation. I made two punctures in each arm, each of which fortunately succeeded, and she regularly passed the disorder, complaining only on the tenth and eleventh days, when the areola was most extended, as is usual. I saw her very frequently during the progress of her disorder. and once or twice after its complete termination: I therefore can speak positively, that during that time she labored under no symptom but what is connected with the cow-pox. From this period she continued perfectly well, and on Saturday last, the 11th instant, she was delivered of a female child, having at the time of its birth many eruptions on it, bearing much the appearence of small-pox in the early stage of the disease. This event happened five weeks after her vaccination, and one month after she had been exposed to the variolous infection of her own three children, and that of several other persons in the same village. On the 14th I visited the child again, when I found the eruptions had increased to some thousands, perfectly distinct, and their character well marked. Many among the most respectable physicians and surgeons from Totness, Ashburton, and the neighborhood, were kind enough, at my request, to come to the poor women's place of abode; and witness the fact. But to put the matter beyond all doubt, I armed some lancets with the virus, and produced the small pox by inoculating with it. On the 18th the infant was seized with slight convulsions, and on the morning of the 19th, it expired."

The following case is related by Dr. Mead, in his discourse on small-pox. " A certain woman, who had formerly had the small-pox, and was now near her reckoning, attended her husband in the distemper. She went her full time, and was safely delivered of a dead child. It may be needless to observe, that she did not catch it on this occasion; but the dead body of the infant was a horrid sight, being all over covered with the pustules; a manifest sign that it died of the disease before it came into the world."—*Amer. Jour. of the Med. Sciences.*

15. *Sulphurous Fumigations.*—In pursuance of the object stated in our last, we commence with an account of the trial made by Dr. Bardsley, at the Manchester Infirmary, of the sulphur Bath, in diseases of the skin, and of some other textures. The extreme obstinacy of a large number of the diseases which pertain to the cutaneous tissue,—their ordinary connection it is difficult to remove, and if removed, still more difficult to avoid in future, and the length of time they have generally existed before application is made for relief, render it very desirable to add some safe and effectual remedy to those already in common use among the faculty. In no instance within our knowledge, has so thorough and extensive an experiment been made of sulphur fumigation as a means of curing such diseases, as that an account of which is contained in the report of Dr. B. Within the last five years, more than 3000 patients have been subjected to the same remedy, and the number of baths given to each has varied from 10 to over 300. The report sets forth, in satisfactory detail, " the *happy results* attending the practice." Bad effects have sometimes ensued from its employment, but most of these are attributed to inattention in the selection of cases,—or to the co-existence with the cutaneous affection of some pulmonary or gastric disorder, leucorrhœa, or other complaint, which may be aggravated, rather than relieved by it.

On the other hand, there are some derangements of other organs in the skin, which, so far from forbidding the use of the sulphur bath, invite us strongly to its employment. In some obstinate cases of *amenorrhœa*, the discharge was found, by Dr. B. to return during the use of fumigation. In like manner has it proved curative of *sciatica, local palsy, scrofulous affections of the joints, obstinate ulcers af the lower extremities, indolent tumors, glandular enlargements,* and other complaints of chronic character. Acute diseases were generally found to be aggravated by it.

In two cases of *Diabetes*, a faithful trial of the sulphur bath was made by Dr. B. In one of these cases, the patient, a young man of 24, had labored under diabetic symptoms for more than six months, and for the last nine had been under treatment at the hospital La Charité at Paris. He made 26 pints of urine daily. After a short course of laxatives, he was subjected to the sulphur bath every second day; and it is equally surprising and gratifying to see, by the table annexed to the case, the uniform diminution of this quantity, which was effected by the treatment. December 2d, it was 26 pints.—February 25th, it was but two pints. On the 17th of March he was discharged in the enjoyment of good health, and returned to his native France and to his former occupation as a manufacturer.

Of 40 cases of *Chronic Rheumatism* from two months to twenty-four years duration, 30 were cured, 7 were relieved, and the remaining 3 were obliged to discontinue the bath.

The particular diseases of the skin in which it was employed with success by Dr. Bardsley, are—

1. *Scabies.*—In this, the sulphur bath is universally acknowledged to be the most elegant and least disagreeable remedy, and Dr. B. found it by far the most successful. "I have always seen, says he, the most obstinate and neglected cases of it yield very speedily to a few fumigations." To this we can add the testimony of our own experience—having never failed to cure the disease effectually and speedily by a few baths properly and thoroughly administered.

2. *Impetigo.*—In this very common and troublesome affection, which resists, as most other cutaneous diseases do, the long catalogue of popular or rather vulgar remedies, as sarsaparilla, dulcamara, pyrola, dock-root, &c., very marked advantage resulted from the sulphur fumigation. It should never be employed, however, until any signs of acute inflammation in the skin have been subdued by leeches and fomentations locally, and general depletion in the few cases in which such resort is indispensable. In that species of the disease termed by Willan Impetigo Scabida, this remedy has proved especially beneficial.

3. *Porrigo.*—In most of the forms of this disease, the bath was found useful. But as it usually occurs in situations where the fumes cannot be conveniently retained, and readily yields to other treatment, it can scarcely be considered as worth while to give it a trial.

4. *Prurigo.*—In whatever form or situation it appears, no local remedy is so agreeable to the patient, or so sure to remove his disease, as that now under consideration. But be it this or any other, the use of gentle aperients and cooling diet is alike indispensable to a permanent removal of the disease.

5. *Lepra.*—Of 40 cases of leprosy treated at the Infirmary by the sulphur bath, 29 were cured, and eleven more or less relieved. To the use of this remedy in lepra and other *scaly* diseases, the author seems particularly desirous of calling the attention of the profession. "My experience of its high value," says he, "leads me to recommend it as a remedy infinitely superior to every other in those obstinate affections." Besides the cases of lepra above referred to, Dr. B. gives an account of four, which occurred in his private practice, in each of which a cure was effected by the sulphureous fumigations *alone;* although all of them were confirmed cases. In his hands this remedy was equally successful in that which is among us the most common of all cutaneous diseases, viz.

6. *Psoriasis.*—20 patients, suffering under this disease, received the bath at the Infirmary. Of these 14 were perfectly cured, 5 were relieved, and I found it necessary to discontinue it.

In addition to these he says, "I have the authority of an eminent member of the bar for stating, that after an *effectual* trial of various remedies, both in London and the country, for the removal of an obstinate psoriatic disease, he obtained a cure by a persevering employment of the sulphur bath."

7. *Icthyosis.*—This disease, most fortunately of rare occurrence yielded to the fumigations, and to these alone, in the practice of Dr. B, He appears, in this and other scaly affections, to have given a full trial to ordinary remedies. Where the bath was used simultaneously, with these medicines, the complaint yielded no more readily than where the fumigations alone were depended on. Still we should deem it unwise to trust to their sole agency in all cases, or to expect, in private practice, that so large a proportion as he has given, would yield to such treatment. The persevering use of acknowledged alternatives, should, in our opinion, accompany that of the bath. Of the two, Dr. B. appears to have found the unaided efforts of the former much less successful. than the latter when administered alone.

8. *Pompholyx.*—The bath was adopted in only two cases of this disease, and in both it proved serviceable. As an example, we will offer the details of one of these cases in the author's own words.

John Smethurst, 15 years of age, was admitted as an out-patient of mine on the 13th of June, 1826. His mother stated that the vesications made their appearance shortly after bathing, whilst the body was much heated from violent exercise. He was first seized with rather severe febrile symptoms, which were removed by the aid of venesection, purgatives, and diaphoretics. The vesications were chiefly confined to the inferior extremities, though many were present on the face and trunk. With a view to their removal, I directed my chief attention to the state of the boy's bowels, prescribing Plummer's pill at bed time, and a gentle aperient in the morning. During the day, he used the decoctum sarsaparillæ compositum, and entered the warm bath every other night. This plan was steadily pursued for more than five weeks, but with very little benefit; for no sooner had some of the bullæ discharged their lymph and healed, than others either reappeared in their place, or arose in fresh parts of the body. I now directed him to enter the sulphur bath every morning, and to discontinue his former remedies. Smethurst received fifty-four baths, when he was discharged perfectly well.

In the 17th volume of the Edinburgh Medical and Surgical Journal, a case of pompholyx is related in the report of the Royal London and Westminster Infirmary for Diseases of the Skin, which was cured by the use of the sulphureous vapor bath. One among many remarkable and instructive cases (says Dr. Emerson), the medical officers wish to bring under your notice. It was an inveterate instance of the pompholyx, which had resisted the various treatments of several hospitals. It occurred in a young girl. Her sufferings were not to be described, but by the use of the bath and some internal medicines, she now enjoys a comparative degree of health and comfort; and her tender age renders it extremely probable, that the cure may be as permanent as it has been satisfactory."

It appears to us that the great fault among general practitioners who have assayed this remedy, has been a want of perseverence in its use. Ten or a dozen baths are considered a fair trial in almost any case; but in truth there are few, very few, instances of disease so mild as to yield to so short a series. In ordinary cases the itch may be cured by this summary process; but with this exception, we should not dare promise amendment even, till twenty had been tried, or think of being discouraged if fifty failed of conquering the disease. The great secret of the success of Dr. B., is his perseverance in the use of this remedy; and those, we apprehend, who follow him in this respect, will be most likely to be rewarded by similar results.

The next difficulty in the way, is the careless manner in which the remedy is administered by unprofessional persons. Women who know nothing of the nature of the disease to be cured, and little more of the nature of the remedy, are, or certainly have been, entrusted with the management of these baths. This is very wrong. It is a powerful application—requires judgment—carefulness—knowledge. "It must be recollected," says Dr. B., "that this method of cure requires both caution and discrimination;" and which of these can be expected in a female who is ignorant of the disease, the remedy, and its modus operandi.

Another obstacle in the way of a full and frequent use of this bath is, the want of a convenient apparatus for administering it. The old sulphur baths were clumsy and inconvenient, and the profession and the public are greatly indebted to Mr. Wallace, Surgeon to the Skin Infirmary in Dublin, for his improvements on the machine. But even these have still their imperfections.

Deterred chiefly by the foregoing circumstances from advising sulphureous fumigations so frequently as we desired in private practice, we caused, a few years ago, a neat and extremely convenient apparatus for this purpose to be constructed; and having since been in the habit of applying it personally to a large number of patients, we have fully tested its advantages over those, more bulky and expensive, which come to us from abroad. We are all, it is true, apt to appreciate too highly the results of our own industry and our own ingenuity; but when those results have been put to the test of direct and repeated experiment, we may be allowed to judge of their value with a greater degree of accuracy. Having enjoyed these facilities, then, for the employment of sulphur fumigations,—moved from the objections to their use, which have already been alluded to, we can readily accord to the accuracy of the observations of Dr. B., although we have seldom employed them to the same extent in any individual case, or found them equally capable of removing chronic and long and obstinate cases of lepra and psoriasis without the aid of internal remedies.

To those who are desirous of further information on the subject of this bath and its value as a medical agent, we recommend the short essay of Sir Arthur Clarke, and the observations of Mr. Wallace—*Boston Med. and Surg. Journ.*

NEW PESSARY.

*To the Editor of the Boston Med. and Surg. Journal.*

16. *Sir,*—I was favored a few days ago, with the 1st No. of the N. Y. Medical Journal. After reading Dr. Beck's Address, Dr. Avery's paper upon the Professions in England and France, with his very illiberal remarks upon Mr. Brodie (for which he is repaid in the Re view of his work on Dyspepsia), I next came to a well written practical Essay on Prolapsus Uteri, by Locke Barber, M. D., &c. &c., read before the N. Y. Medical and Philosophical Society,—a disease which gentlemen in extensive obstetric practice often meet with, and as often fail in giving permanent relief, without the aid, either of the natural pessary of pregnancy, or one made of the many substances, compositions, or shapes, suggested by different authors, ancient and modern. Wood, silk, linen, elastic gum, cork-wood, box-wood, ebony, steel, silver, and even gold, have all had their advocates in turn. The round, oval, flat, globular, and so forth, have been equally praised for shape. Towards the latter part of Dr. B's Essay, he strongly recommends a globular air pessary, of which he appears to consider himself the inventer. He thus expresses himself:—"The instrument which has been alluded to for supporting a prolapsus of the womb, and the one which *I have had the honor to introduce into practice*, is of a globular shape. A bag of elastic gum, of a suitable size, is secured, perfectly *air-tight*, to a tube of ivory and silver. The tube contains a valvular mechanism, capable of retaining such quantity of air as may be introduced into the bag, which is done by means of other tubes, and a large elastic bottle, so that the pessary may, with the greatest ease, be either inflated or exhausted, after it is introduced into the vagina. By a reference to the plate and explanation, a better idea of its principle may be gathered, than from any other description. In 1829, Dr. Painhaud, an intelligent and extensive obstetric practitioner of Quebec, read before the Quebec Medical Society* a paper on Pessaries—an extract of which I have obtained in the words and language of the author.†

"You will recollect, gentlemen, that at the close of the last paper which I had the honor to present to this Society, reference was made to a new pessary which I had invented. Having tested its value by subsequent experience, I propose this evening to explain to you its peculiar construction. The subject is one of much more importance in practice than is usually accorded to it." * * * *

"In surgery, as in other arts, we are all influenced by a love of invention, but the great error is that we are too ambitious—we are emulous of splenpid discovery, and disdain to give our attention to subjects of small pretention." * * * * In all sciences, discoveries, often the most important are the result of accident; and it is to accident alone, that I am indebted for the pessary I am about to describe."

---

* The Quebec Medical Society has been in existence some years,—papers of considerable merit remain on their shelves unpublished; and if asked why, I should be at a loss to answer

† This extract was sent us in French—the language in which the paper was originally written and read to the Society. In order to save the reader some trouble, we have hastily translated it into English, preserving certainly the facts, if not the precise diction of the author. Our correspondent will excuse us for omitting part of the extract sent. On examination he will find it irrelevant to his purpose.—Ed.

"A few months ago, I was present at an operation, by a professional friend, for *fistula in ano.* Hemorrhage became alarming, and not being able to apply a ligature, or sufficient pressure to arrest the bleeding, the operator directed compresses wet in iced water to be applied constantly. It occurred to me to introduce into the rectum an empty bladder, of small size, and then to fill it with cold water by means of a syringe: I then drew it outward, (having secured its neck) until it pressed against the bleeding vessels with force sufficient to arrest the hemorrhage; and thus was the life of the patient saved. Reflecting on the possibility of applying this remedy in other and often dangerous cases of internal hemorrhage, the idea of its use as a *pessary*, suggested itself to my mind; and in the *bladder of a sheep*, I found a pessary of a construction heretofore untried, and of a material analagous to that of the parts with which it is to lie in contact; an instrument readily obtained; easily introduced when empty of air; easily removed by letting out the air, water, or other liquid with which it had been injected; easily retained in its place, as it becomes larger than the passage through which it was introduced; and worn without pain to the patient, so similar is its material to that of the part on which it rests, it is scarcely possible, in fact, to find in any instrument a more perfect adaptation to the purposes for which it is designed."

Having communicated this discovery to Dr. Morrin, that skilful practitioner thought it deserving the attention of the profession, and proposed as a substitute for the bladder, the gum-elastic bag, such as is used for injections in cases of Hydrocele."

Being present at the reading of the above paper, I understood Dr. Morrin to say, in addition to his suggestion about the hydrocelle bottle, that one made of the same substance, but circular and hollow, to be inflated in the same manner, would, he thought, be preferable to either, inasmuch as it would not necessarily have to be removed during menstruation; and concluded by regretting he was going to contest Dr. B's claims as the inventer of the Air Pessary. Opening at the moment the 3d edition of Dr. Aitken's Principles of Midwifery, or Puerperal Medicine, published in London, in 1784, he read as follows:—"Supporting the replaced uterus is not a little difficult. It is attempted; 1st, by a pessary; 2d, bandage.—*Pessary.* Of all the varieties of this instrument, that is preferable which possesses the following qualities,—1st, smoothness; 2d, lightness; 3d, compressibility. I have invented the Air Pessary, which has these characters in a greater degree than any other I know of. The Air Pessary is formed of a small bladder or bag, soft and air-tight, with a valve at the orifice. It is introduced, and then duly inflated, by the patient, by a long flexible tube, which is immediately retired. This instrument, while it is exceedingly light, fully occupies the vagina, and supports perfectly the uterus. When it is wished to retire it, the valve is forced, and immediately it collapses" (see plate xi). During the reading, and for some minutes after, the palsied and astonished countenance of the Dr., at being so suddenly and unexpectedly deprived of his claim as the inventer of the Globular Air Pessary, satisfied every member present that he was no plagiarist, but truly the inventer of what turned out to be an "old friend with a new face." The above, Mr. Editor, is an unvarnished and plain statement of facts, as I became acquainted with them. I should hope there has been no literary pilfering among the competitors.

Yours, &c. *A Quebec Subscriber.*

17. *Efficacy of Hydrocianic Acid in curing Vomiting not dependent upon Inflammation.*—We extract from a clinical lecture by Dr. Elliotson, reported in a late number of the London Medical Gazette, the following remarks on the efficiency of hydrocianic acid as a means of arresting vomiting. It should first be ascertained, says Dr. E., whether there is inflammation or not; for if there be inflammation, the hydrocianic acid would not cure it; the case must be treated like inflammation of any other part of the body. But if you can find no inflammation whatever, and you find no cause for vomiting in any other parts of the body, (it will often arise from an irritation in the intestines, the kidney, the womb, and ten thousand distant causes,) then the hydrocianic acid will relieve the vomiting far more, I am satisfied, than any other medicine. I have not found it relieve the pain of rheumatism or cancer, or pain situated in any of the distant parts of the body, or pain in the intestines; it is of no use in colic, though it is said by some to be of occasional service in neuralgia. As an anodyne, I have not found it of the least use in general, except in cases of pain of the stomach. It has the properties of an anodyne on the stomach particularly, and has a tendency to lessen the morbid irritability which produces vomiting. It is no exaggeration when I state that I have frequently seen vomiting, which has lasted for months, cease on the exhibition of the first dose of this medicine. Frequently however, in cases of spasmodic pain of the stomach, you will find that the first dose, or the second, or even one week's exhibition, will not answer the desired end; you will be much more struck with its use in lessening vomiting than in lessening pain in the stomach. But you will find it of no service unless you make a distinction between the existence of inflammation and the influence of distant causes on the one hand, and mere morbid irritability of the stomach itself upon the other.

Hydrocianic acid is a medicine that is exceedingly powerful, and you cannot give it in the same dose when the stomach is empty as when it is full. When the stomach is full, the difference of a drop may cause a great difference in the effects. Supposing you are giving three drops, three times a day after meals, it certainly will not be right to give more than one or two drops upon an empty stomach. To avoid any confusion which may arise, it is best always to give it after meals, otherwise you must vary the doses at different times of the day. You cannot, in general, give it on an empty stomach more than once in the day, because when food has been once taken, the second meal comes usually before the stomach is as empty as it was before. On this account I make it a rule to give it after breakfast, in the aftenoon, and the last thing at night. As it is so powerful, you cannot tell beforehand the dose that will be borne, and you should begin with a small quantity, such as you know can hardly disagree with the stomach. I begin with one minim, though you may begin with two, and many persons do so, but it is safer to begin with one. I give one minim three times a day, diluted with water, or aromatic water; and in the course of a day, if no unpleasant effect be produced, I increase the dose to two minims, and the third or fourth day I give three minims, and so on till it produces the effect I desire, or some inconvenience arises. Although it will relieve the vomiting arising from mere morbid irritability, it will, from its irritating properties, likewise cause it. If you give an over dose, it may produce extreme nausea, extreme vomiting, and perhaps gastrodynia—pain in the stomach. It is common for many narcotics to be stimulating as well as sedative, and this is the case with this medicine; and medicines act with different power upon different people; and therefore you should give it in small doses at first, if you wish to make it act favorably. Tobacco will arrest the action of the heart, and cause complete prostration of strength, and yet it excites sneezing, and one person is effected by a quantity not noticed by another. In general, people bear from two to four minims, but you not unfrequently meet with individuals with whom five minims do not disagree, and now and then you may safely increase the dose to six or eight, or even more.

You will find this of great use for another purpose—for making other medicines sit upon the stomach, which would otherwise disagree with it. You may lessen the natural irritability of the stomach so much, that iodine, colchicum, and medicines of that active description, will frequently sit upon it, whereas they would not, unless, ten minutes before you administered them, you gave a dose of hydrocyanic acid. These medicines may remain upon the stomach if you unite prussic acid with them, but it answers better for this purpose if given ten minutes before, so as to come into full operation before the acrid medicines are taken. In organic affections of the stomach, you will frequently find it better than any other medicine; if there be cancer of the stomach, schirrhus, pylorus, or whatever the organic disease may be, you will often find the hydrocianic acid allay the vomiting and pain much more than any thing else.—*Gazette Medicale.*

18. *Radical Cure of Hernia.*—We find in the 'Gazette Medicale de Paris,' for July last, a report by M. Breschet, to the Academy of Medicine, on two memoirs by Dr. Ravin, of St. Vallery-sur-Somme, on the radical cure of hernia. This method is only applicable to recent and small, or better to old and large hernias, in which there are no adhesions between the sac and the contained parts, and in which the latter is perfectly reducible. The method consists in reducing the hernia, and keeping the part which protruded as long as possible at a distance from the canal, so as to allow of the slow contraction of the aponeurosis, dilated by the protrusion of the intestine, which concontraction, M. R. says, will take place when no mechanical obstacle is opposed. The method of M. R. requires that the patient should be placed upon his back, the pelvis raised higher than the diaphragm, and the side on which the hernia is situated hgher than the sound. A bandage must be placed over the orifice of the ring, and compresses, either dry or moistened with tonic and astringent lotions, placed between the cushion of the bandage and the inguinal ring; the most absolute rest is also necessary.

M. R. relates eight cases cured by these means. The cases occurred in persons of different ages, and both sexes. Thus one was a man, aged twenty-six, with a recent and small inguinal hernia—he was cured in twenty-six days. Another, a woman aged sixty, affected with hernia for upwards of twenty years, was cured in six months. The ordinary duration of treatment, however, has been two months. M. R. requires his patients to wear a bandage for some time after their cure, but he has never seen a relapse except when the patient has not continued in a recumbent posture during the whole course of the treatment.

The reporter speaks favorably of the candor with which the memoirs are written, and the evident desire of the author to relate the truth, and to advance science. The treatment is safe and worthy of trial.

M. Dublas, of Lyons, has also communicated to the Academy of Lyons, a memoir on the radical cure of hernia; the method proposed by him is:—diet, repose, recumbency on the back, and astringent applications upon the hernia. The Gazette Medicale, to which we are indebted for this notice, does not furnish us with further details.—*Amer. Jour. of the Med. Sciences.*

19. *New Method for the Cure of Hernia.*—M. Belmas has communicated to the Institute, a method for the cure of hernia, proposed by himself, and which is thus described in the *Journal des Progres,* Vol. III. 1830. If a gold-beater's skin filled with air, be introduced into the abdominal cavity of a dog, it excites around it, but merely to an extent corresponding with its size, an inflammatory irritation, followed by a lymphatic deposition, an absorption of air, and also of the bag itself; so that at the expiration of some days, a concrete albuminous mass can only be found, and after some weeks only a fibrous knot remains. Similar experiments made by M. Belmas on the hernial sac of dogs, have been followed with the same result as in the abdominal cavity. Filled with the distended bag, the sac inflames through all its extent, and is obliterated in a few days in the manner above-mentioned, without preserving any remains of the foreign body introduced. Of thirty female dogs affected with hernia, which have thus been operated on, in spite of the indocility of these animals, thirteen have been radically cured. M. Belmas operates in the following manner:

A small bag of gold beater's skin, perfectly empty, is connected with a tube furnished with a stop-cock. A small incision is made with a short and narrow bistoury, at the most inferior part of the hernial sac, the edges of which are drawn asunder, and there is introduced into the sac a canula and stilette, to the extremity of which the tube of the bag is fitted. The point of the canula is pushed with suitable precautions to the superior part of the sac, near the neck. The stilette is then protruded, and through the opening which it makes in the skin, the cannula and the tube of the bag are to be drawn out. To this tube is to be attached that of another bag filled with air; the stop-cock of the first is to be opened, and a sufficient quantity of air is injected into the bag of gold beater's skin, and there retained by closing the cock. The operation has already been practised on a man, and with complete success, notwithstanding unfavorable circumstances, such as the advanced age of the patient, his cachectic constitution, a paralysis of the bladder, and a chronic catarrh. Much swelling succeeded on the second day, and M. Belmas dreaded some unpleasant consequences, but none occurred. For two months and a half there has been no return of the hernia, although the patient is the subject of an habitual cough. This man has been presented at the clinique of the Hotel Dieu.

It should be remarked, that the bag should be introduced, in a dry state, into the sac. M. Belmas, who at first thought it most convenient to cover it with oil, discovered that this alone was sufficient to prevent its absorption.—*Ib.*

20. *Cases of Dislocation of the Thigh Bone, with Remarks, by the late Mr. Averill, Surgeon, Cheltenham.*—Case 1.—*Dislocation of the Thigh Bone into the Ischiatic Notch.*—On Tuesday evening, September 2d, 1828, I was called to the hospital, to see Thomas Perry, 6 1-2 years old, whom I found in his father's arms, struggling and crying very loudly. He was stated to have received an injury to the lower part of the belly and the thigh, by a person jumping accidentally upon him out of a rail-way cart, and alighting between his thighs. As I perceived he moved both his legs, and as they appeared both of the same length, I conceived it to be only a bruise, and directed him to be put to bed for the night. The following day there was considerable swelling round the hip, and handling the limb occasioned the child a great deal of pain, and caused him to cry loudly. An evaporating lotion was directed to be applied, and aperient medicine was ordered. The next day, the swelling having still increased, leeches were applied, and any attempt to move the limb, or make a particular examination, set the boy crying to such a degree as almost to cause convulsions. Occasionally, during the one or two following days and nights, he cried so much as to disturb the other patients, and the nurse observed he started frequently in his sleep. As the swelling subsided, he became quieter, took his food, and appeared easy. To-day, the ninth day from the receipt of the injury, the swelling having quite subsided, I made a more particular examination of the limb, and found that the head of the right thigh bone was dislocated into the ischiatic notch. On placing the limbs parallel to each other, and placing the toes together, the dislocated limb appeared to be from half to three-quarters of an inch shorter than the other; and the knee was inclined inwards; the limb admitted of rather free motion, and when it was rotated the head of the bone could be felt behind the acetabulum lying over the ischiatic notch.

My colleagues having examined the limb, and agreeing with me as to the nature of the injury, it was considered best to attempt the reduction with the pullies. To effect this, a wetted roller was applied above the knee, to which another portion of doubled roller was attached; the pelvis was fixed by a silk handkerchief being passed between the thighs, and then attached to the upper bed-post. The pullies being fixed to the roller at the knee, and attached to a table placed against the foot of the bed, another handkerchief was passed round the upper part of the the thigh bone, so as to lift the head of the femur over the margin of the acetabulum. Extension was made in a direction obliquely forward, and kept up for twenty minutes, when the knees appearing parallel to each other, the pullies were suddenly loosened, in the hope that the head of the bone would be drawn into the socket; this however, was not the case, for it returned again to the ischiatic notch. Extension was again made, and continued for near half an hour, when, at the time the knee was being rotated outwards, the head of the bone was drawn into its natural situation, the sudden jerk of which, though in so young a subject, was evident to all assisting in the reduction. The knees and feet were confined by a roller, and the patient was left.

Sept. 23d.—To-day he was lifted from his bed, placed on his feet, and allowed to try to walk, which he could not do without support. I directed that he might try the same daily.

Dec. 4th.—He was discharged from the hospital able to walk with but little lameness.

In offering a few observations on this case, I must plead guilty to the charge of not having minutely examined the boy when first admitted into the hospital but I had no suspicion of a dislocation occurring in so young a subject, and I felt at the time satisfied, from the degree of motion of which the limb admitted, that the part was only bruised. That a dislocation into the ischiatic notch, in so young a subject as the one here related, is a very rare case, may be inferred from the fact that Sir A. Cooper. in the course of his extensive practice, has never met with an example of it. He has related one case of a little girl seven years old, in whom the head of the bone was dislocated on the dorsum ilii, and this is the only case he has known of dislocation from accident in the young subject. When much swelling and inflammation are present, to make out the exact nature of injuries in the vicinity of the hip joint, is exceedingly difficult, and, in all cases, not possible. I was once consulted in a case of this kind, in which, not exactly agreeing, as to the nature of the injury, with the gentleman who was in attendance, a third was called in, of greater experience than either, and who differed from us both, giving his opinion in the most decided manner; yet this gentleman, I understand, on seeing the case again, after the tumefaction had subsided, gave an opinion directly opposed to that he had previously so unequivocally expressed. A further proof of the difficulty of making a correct diagnosis of injuries in the neighborhood of the hip joint, is evinced in the case of a physician of eminence in an adjoining county, who, I am informed, though a long sufferer, and still lame from an accident of this kind, is not aware of the injury he has sustained. No means of reducing the muscular power were resorted to, in the above case, further than exhausting it by continued extension. The boy was but a weakly subject, and I did not choose to risk the chance of a fatal termination, by producing a state of exhaustion from which it was possible he might not have recovered.—therefore, neither the warm bath, nor emetic tartar, were had recourse to. My fears on this head must not be considered wholly without foundation, as two cases have come to my knowledge, in which death has speedily followed that state of collapse, produced by these means of depletion which are occasionally had recourse to, with success, during the attempts to reduce dislocation of this description.

CASE II.—*Dislocation of the Thigh Bone into the Foramen Ovale.*—On the night of August 5th, 1829, I was sent for, with Mr. Skelton, of Charlton, to see Mr. Acott, aged 21, a muscular farmer, who, in returning from Cheltenham fair in a gig, with two others, was thrown from the vehicle, in consequence of the horse taking fright. We found him lying on his back, in bed, with his right thigh separated widely from the left, and the extremity near two inches longer than the other, admitting of very little motion, and causing great pain. Though the head of the thigh bone could not be felt, there was no doubt in the minds of Mr. S. or myself, that it was dislocated into the foramen ovale, and, as soon as the proper apparatus could be procured, we reduced it in the following manner:—The patient was placed on his back, on a table, in the passage of the house; a jack towel was carried round the pelvis, to fix it, and a belt, to which the pullies were attached, were passed round the upper part of the thigh, between it and the scrotum, and fixed to a staple in the wall. About twenty ounces of blood were taken from the arm, and a grain of emetic tartar given; slight nausea was soon produced; extension was then made in a direction obliquely outwards and upwards, and, in about three minutes, on bringing the knee inwards, the head of the bone was drawn suddenly into the acetabulum. The feet were then bandaged together, he was put to bed, and in about ten days left his room.

The ease with which this dislocation, in a very muscular man, was reduced, forms a striking contrast to the preceding, and shows how desirable it is that the attempt to replace the bone should be made as soon as possible after the receipt of the injury, and before the muscles have become in a permanent state of contraction.

CASE. 3.– *Dislocation of the Thigh Bone on the Dorsum Ilii.*—Nov. 24th, 1829.—In consultation with Dr. Boisragon and Mr. Seager, I saw a lady, aged 44, who, a few days previously, had dislocated her right hip. As the patient was thin, the head of the bone could be felt on the dorsum of the ilium; the limb was shortened, so that the great toe was resting against the tarsus of the other foot. As an attempt to reduce the dislocation the day previously had been made, with towels, we thought it better to employ the pulleys, which were used in the precise manner directed by Sir Astley Cooper, for the reduction of this kind, and it was not till extension had been continued for upwards of an hour, that the head of the bone was replaced, which happened while the knee was being rotated. Emetic tartar was administered to the extent of eight grains, previous to, and during the reduction, but no sensible effect was produced by it, till upwards of two hours after she was replaced in bed. The feet were bandaged together, and she did very well.

Our efforts to reduce this dislocation were considerably opposed by those of the patient herself, who was, at the time, insane, to which circumstance the tardy action of the emetic tartar may properly be attributed.—*Midland Med. and Surg. Reporter.*

21. *Remarks on the use of Injections of Warm Water, with a Description of an Apparatus for their Administration.*—By Leonard Pierce, M. D. of Sutton, Mass.—In the Boston Medical and Surgical Journal, of May 3d, I read some remarks on the use of injections of syringe used for that purpose. I have been in the practice of using these injections for different diseases, especially the spasmodic ones, for more than two years past, and have been pleased to find them producing the most happy effects. Most of the cases in which I have used these injections, have been of colic, convulsions, and constipation. The quantity of water thrown up, should be from one to four quarts, according to the effect intended to be produced. The common injection syringe is inconvenient for this use, from the necessity for its frequent application. To remedy this evil, I had a syringe made by Mr. Oliver Hall, an ingenious artist of this town, in the form of a pump, with a pipe at the end for admitting the water, guarded by a valve opening inwards; and another pipe at the side to be attached to a tube, communicating with the rectum, with a valve opening outwards. This latter valve has a spring on its outer surface, by which it is kept constantly closed except when forced open by the descent of the piston. The body of the syringe, the pipes, and the cap, are of tin (block tin); the shaft of the piston is iron, coated with silver to prevent its rusting; the piston itself is a cork turned to fit the calibre of the pump, or syringe (which is about three-fourths of an inch in diameter), and confined between two plates, the upper one of brass, the lower one of iron, through the centre of which the shaft passes. The lower plate, or that nearest the end of the shaft, screws on so that the cork may be compressed, and its diameter increased whenever necessary. The whole length of the pump, when the piston is down, including the end pipe and the elevation of the shaft above the cap, is between five and six inches. To the end-pipe a wooden tube is fitted, with holes through the sides, near the lower end, to let the water through. This tube, when used, may be placed on the bottom of the vessel containing the water, and thus serve to support the pump. It may be dispensed with at any time, if found inconvenient. The common flexible stomach tube is the one I use for connecting the pump with the rectum. To the open extremity of this tube, a piece of wood is attached, into which the pipes are made to fit. The pipes are made so as to be separated from the body of the pump, in order to get at the valves, should they need repairs.

The whole of the apparatus, except the tube, may be packed into a box and carried in the coat pocket. The tube might be made in pieces connected together with silver, so as to render it suitable for an œsophagus tube, and the pieces be packed in with the pump.

When it is to be used, the patient should be placed upon the bed on one side, with his back near the edge of the bed. An assistant should then take the tube, and having it well anointed with lard or sweet oil, introduce it into the rectum from two to twenty inches, according to the effect intended to be produced. When the intention is merely to solicit an evacuation, it need not be introduced farther than within the spincter ani, unless it be in obstinate cases of constipation, when it should be put far up. If the tube meet with any impediment to its passage, attach the pump, and throw up some water, which will remove the obstruction if it be hardened feces. If, after this has been done, the tube should not pass up readily, or if the patient should complain of much pain when the attempt is made, it is presumed that the obstruction is a spasm of the intestine. In this case, more water should be gently pressed up, occasionally making an attempt to carry up the tube. This will generally succeed in overcoming the spasm, so that the tube may pass and the injection be completed.

Generally, after from one to two pints have been thrown up, vomiting comes on, after which, in cases of colic or convulsions, the disease abates, and the patient falls asleep. But in this case the better way is to let the tube remain in the rectum; for frequently the pain and convulsions will return, and more water will be required. Perseverance in this course will almost always effect a cure, without any other remedy than a little opium after the spasm or convulsions are overcome. In violent cases, bleeding may be necessary; it should always precede the injections where an inflammatory action is established before they have been given. This treatment does much less injury to the intestines and the system generally than cathartics—the relief is altogether more prompt, and a relapse is much less to be feared.

These injections are often very efficacious in the treatment of obstinate cases of constipation. They should be given at stated periods, and in pretty large quantity, so as to pass into the colon, and, if possible, into the small intestines. I think they might also be used with advantage in some of those cases usually called dyspepsia. In cases of hysteria attended with convulsions or with coma, they must be useful, although I have never given them a trial. In the incipient stages of cholera and dysentery, soothing injections, such as mucilage of gum arabic, flaxseed tea, starch, &c., are very useful, and in many cases will be rendered more so, if thrown up in large quantities.

In no case in which I have used these injections, have I combined any other ingredient with the water than a little molasses, sufficient to give it the color of brandy. I have seldom, however, made this addition; but from the effects in those cases where I have used it, I think it will be useful where it is desirable to produce a determination to the skin.

The most immediate effect produced by these injections, is vomiting. In every case where I have used them to the extent of two pints or more, vomiting has followed almost immediately, and I believe it to be the most speedy, effectual, and least irritating method of evacuating the stomach that can be adopted, unless it be by passing a tube down the œsophagus. Stimulants are seldom if ever useful where a large quantity of water is used. In the 14th No. of the American Journal of Medical Sciences, is a case related by S. C. Roe, M. D., of New-York, in which the patient swallowed two and a half ounces of laudanum, with an intent to kill himself, and obstinately refused to take any remedy. Dr. Roe introduced into the rectum the tube of a stomach pump, twenty-three inches, and threw up half a gallon of water, holding in solution fifteen grains of tartarized antimony. "This," says Dr. Roe, "was no sooner accomplished than the patient complained of nausea and an inclination to evacuate his bowels, which was quickly followed by full vomiting, repeated several times successively; but the disposition to alvine dejections passed off without effect." It will not, I believe, be contended, that the medicines introduced into the intestines, with an intention of producing an effect on the stomach, will act more promptly than when taken into the stomach itself. But according to Dr. Roe, the medicine, which, when taken into the stomach, requires from twenty to forty minutes to produce its full effect, operated almost instantly, and most effectually too. Now this is precisely the effect which I have witnessed in numerous instances where nothing was used but water, or molasses and water, and I believe the only effect of the antimony was to increase the "languor and debility" of the succeeding day.

Injections of cold water would probably be useful in the incipient stages of inflammation of the bowels. But after the intestines have become seriously diseased with inflammation, it may be questioned whether they would be beneficial. I used them in but one case of this kind, and that was where the disease had existed several days, and an inflammatory action was established. The pain occasioned by throwing up the injection was so great, that I was obliged to desist. It was probably produced by cold, and distention of the intestines. The injection used was flaxseed tea. In all cases where injections are used, great care should be taken not to force them up too fast; for after having distended the intestine, it might and probably would be ruptured, if much force should be applied to the piston. Every person acquainted with the mechanical properties of fluids, knows when a closed vessel is filled with a fluid through a small opening, the pressure on the inner is as much greater than the pressure of the fluid through the tube, as the area of the vessel is greater than the area of the opening. It is by virtue of this law, that a man standing on the board of a collapsed bellows, and having a small tube passing through the board to the inside of the bellows, all other openings being closed, can, by blowing through the tube, fill the bellows with air, and raise the whole weight of the body merely by the power of his lungs. This must render it evident to every person, that there will be great danger of rupturing the intestines, if much force be used in forcing down the piston. Nurses are not only generally ignorant of this law of fluids, but suppose that the larger the vessel, the more divided the pressure will be, and that hence the more water is thrown into the intestine, the less caution will be necessary, and, instead of abating, will increase the force upon the piston of the pump. They should always be strictly charged on this subject, when entrusted with the administration of enemata. Where circumstances do not render it improper, the physician had better throw up the injection himself. Having the patient placed on the bed as directed, the tube may pass out from under the bed-coverings, and the pipe be attached to it, and the injection given while the patient is not in the least exposed. If this method of administering enemata were generally adopted, and simple warm water, or molasses and water used instead of those loathsome and disgusting compounds which many nurses and some physicians seem to vie with each other in conjuring up, much of the prejudice which now exists in relation to them would be overcome, and many of those active and irritating medicines which are now kept in the family medicine-chest for indiscriminate use, would make way for the much less hazardous, but more effectual remedy,—an injection syringe and its appendages.

This instrument may also be used for a stomach pump, either for filling or evacuating that organ. The tube is made to be attached to either pipe, thus making it a suction for a forcing pump, as occasion may require. An objection may be made to its use as a stomach pump, from the liability of its valves to become obstructed with the contents of the stomach. But I believe but very few if any cases will occur, in which it will be necessary to use the pump for the purpose of evacuation. When the tube is introduced, the resistance of the œsophagus and cardia is entirely overcome, and the evacuation of the stomach through the tube is by the same law it would be if no vitality existed in the parts. Now this being true, if, after the stomach is filled with water, the mouth be placed lower than the stomach, this ocgan will be as surely and effectually evacuated as it could be if the pump were used. Therefore, after sufficient water has been thrown into the stomach, let the patient lie upon the bed with the face downwards, the stomach lying across some pillows placed for the purpose, and the head depending over the edge of the bed. Gentle pressure being now made over the stomach, the water will be driven out through the tube. After all the water has been discharged, more may be thrown in and again pressed out, and in this way the stomach be effectually drenched. Or, if the contents of the stomach be too coarse to pass through the tube, let the water be given per anum sufficient to produce vomiting. Vomiting induced in this way, occasions much less effort, than when produced by an emetic.
*Sutton, May,* 1831.

22. *Lithotrity.*—Some time since, the general administration of the hospitals of Paris, gave to M. Civiale, the charge of a ward in the Hospital Neckar, which was assigned for the treatment of calculous patients by lithotrity. In a report lately made to the Academy of Sciences, and which we find in a late number of the Gazette Medicale de Paris, M. Civiale states, that within the space of six months, sixteen patients were admitted into his ward: seven of these were operated upon by lithrotity, four by lithotomy, and the five others were not in a condition to undergo any operation.

The first case operated upon by M. C. was a young man; the calculus was friable, and filled the cavity of the bladder almost entirely, so that it was difficult to inject into that organ a few spoonfuls of water. The patient was cured.

The calculus in the second patient was small, but there was stricture of the urethra, which it was necessary first to destroy—great tumefaction of the prostate, and catarrhus vesicæ. These circumstances would have rendered the operation impracticable, had the calculus been large; being but small, it was completely destroyed in ten minutes.

The third patient was a man of almost eighty years of age, much worn down by acute and long-continued sufferings. The stone was as large as a pullet's egg. The patient supported the operation well, notwithstanding his advanced age.

The fourth patient was sixty years of age, and so irritable, that the introduction of a bougie into the urethra produced convulsive actions. The irritability was diminished by the repeated introduction of bougies and an appropriate medical treatment, and in four sittings of some minutes each, the calculus, (an uric acid one,) which was the size of a small nut, and very hard, was destroyed. The fifth case was that of an old man, exhausted by pain and the great alteration of the genito-urinary organs. The bladder was the seat of catarrh, and contained a great number of calculi, requiring many sittings for their destruction. The other two cases do not offer any thing very remarkable in their details, except that in the one the patient had learned to arrest the evacuation of his urine before the bladder was completely emptied, and thus preserved himself from the pain commonly suffered by calculous persons when the bladder contracts upon the stone; and the other is interesting as showing the little influence which the operation exerts in general upon other coexisting organic lesions.

M. Civiale states that he has himself operated upon one hundred and fifty-two patients, but he does not communicate the proportion cured.—*Amer. Jour. of the Med. Sciences.*

23. *Section of the Sciatic Nerve.*—This operation has been performed by Dr. Malagodi, of Bologna, for the cure of an obstinate neuralgia, seated in the superficial nervous ramifications of the leg and foot, eleven years duration, and which had resisted a multitude of remedies. Pievious to the operation, Dr. M tried a number of experiments upon dogs, from which it appeared that the section of the sciatic nerve, two-thirds down the thigh, produces paralysis, and subsequently atrophy, but not gangrene. That this paralysis extends from the middle of the leg to the extremities of the toes—that the leg does not, however, lose the power of sustaining the body, or become unfit for the purposes of locomotion, the muscles which move it not being paralysed, and the knee joint not being affected. It is also proved by amputations of the thigh, that the division of the nerve does not compromise life. These results determined Dr. M. to perform the operation, which he accordingly did on the fifth of March, 1828, in the presence of Professors Matheo Venturoli, Paolo Baroni, and M. Joseph Bertolotti. The patient was a man aged thirty-one years. The incision was made about three inches above the popliteal hollow, and was two inches and a half in extent. At the moment when the section of the nerve at the upper part of the wound was made, there occurred a trembling of all the limbs, and a pain similar to the pricking of needles rapidly extending from the place of section to the spinal column and brain, and the patient fainted. When he recovered, which was very soon, Dr. M. requested him to be very attentive to his feelings, and the nerve was then divided at the lower portion of the wound, without the patient being aware of it. Upwards of two inches of the nerve was thus removed, in order to prevent its reunion. From the moment of the division of the nerve, all pain ceased, and had not returned five months after, when Dr. M. communicated the case to the Medico-Chirurgical Society of Bologna. There was paralysis however of the leg and foot, with tingling and sensation of weight in these parts, but there remained an obtuse sensibility of the inside of the leg.—*Gaz. Med. Feb.* 1831, *from the Osservat. Med. XXIII.*

24. *Wound of the Pericardium and Heart.*—M. Larrey exhibited to the Academy of Sciences, at their meeting on the 12th of December last, a soldier who had been wounded by a ball, which passed through the chest, entering two or three lines from the left nipple, and passing out between the spine and the scapula, half an inch from its inferior angle. It is supposed that the ball passed through the pericardium, a portion of the left lung, and furrowed the surface of the heart. There was, at the moment of the accident, extreme prostration, with all the symptoms which characterise these lesions. It was expected, for the first forty-eight hours, that he would expire every moment. By appropriate means he was recalled to life, and the cure was complete.—*Gazette Medicale.*

25. *Vagitus Uterinus.*—M. Baudelocque has communicated to the Academy of Sciences, a case of labor, in which the waters being evacuated, the face of the child presented to the neck of the uterus, and the child uttered cries as strong as if it had been delivered. This and some analogous facts prove, according to M. Baudelocque, that it is important to introduce air within the membrances, as he has proposed when the child is in danger of perishing.—*Journal Universel et Hebdomadaire.*

26. *New symptoms of Pregnancy previously to the Fourth Month.*—It is well known how uncertain the signs of pregnancy are, previously to the fourth month. Dr. Beccaria announces in the *Annali Universali di Med.* for September last, that he has discovered a symptom by which it may be determined: this is a very acute pulsating pain in the occipital region, occupying particularly the part in which Gall locates the organ of the instinct of reproduction. This pain, Dr. B. says, is accompanied with giddiness on the least motion of the head, and with difficulty in supporting the light. This pain comes on suddenly, without any premonition; it continues for some time; an inclination to sleep succeeds, and after sleeping some minutes, the patient awakes completely free from the pain, and with a strong desire for food. These pains re-appear daily, at nearly the same hour, for about eight days; they afterwards disappear spontaneously, without any remedy employed to remove them earlier. This sympton, Dr. B. says commonly manifests itself, unaccompanied with the signs usually laid down as denoting pregnancy previously to the fourth month. Dr. B. asserts that he has observed this symptom in women who were not aware of being pregnant, and even did not suspect the fact.

Should the assertion of Dr. Becaria prove true, it will not only be highly important in a medico-legal point of view, but extremely interesting in a physiological one.

### MEDICAL STATISTICS.

27. *Report of the Coombe Lying-in Hospital.* By Richard Reed Gregory, Esq.—Since the opening of this institution on the 3d of February, 1829, 691 patients have been delivered in the hospital, of whom nine died. The presentations were as follows:—natural 645; face 2; breech 14; feet 7; arm 3; shoulder 1; funis 7; twin cases 12; total 691.

"In two of the twin cases the breech presented, in two others the head; in one the first child presented with the head, the second with the feet; in another the first child with the breech, the second with the arm, the other six were presentations of the head and breech.

"The following table exhibits the number of births, and the sex of the children, arranged according to the age of the mother, from fifteen to forty-five inclusive, taking an average of every five years.

| From | Males. | Females. | Total of Births. | Still Born. | | Total Still Born. |
|---|---|---|---|---|---|---|
| | | | | Males. | Females. | |
| 15 to 20 | 19 | 15 | 34 | 2 | 2 | 4 |
| 20 to 25 | 114 | 93 | 207 | 14 | 6 | 20 |
| 25 to 30 | 100 | 88 | 188 | 9 | 7 | 16 |
| 30 to 35 | 106 | 79 | 185 | 10 | 2 | 12 |
| 35 to 40 | 33 | 23 | 56 | 1 | 1 | 2 |
| 40 to 45 | 15 | 17 | 32 | 0 | 1 | 1 |
| 45 to 50 | 0 | 1 | 1 | 0 | 0 | 0 |
| | 387 | 316 | 703 | 36 | 19 | 55 |

"Of the still born children there were 29 premature, 2 abortions, 3 arm presentations, 3 natural cases, 3 crotchet cases, 4 cases of prolapse of the funis, 3 feet presentations, and 4 breach ditto: of the 12 cases of twins, four children were still born, making a total 55 deaths. Premature labour is a circumstance of very frequent occurrence among the women residing in this district of the city, owing to their irregularity of living, and the brutal treatment they often receive from their husbands.

"Of the arm cases, one did not come into hospital until twenty-four hours after the waters had drained off, and the child had been forced into the pelvis by the violent action of the uterus, which continued after her admission, so as to render turning impracticable. Spontaneous evolution, (as it is termed by Dr. Denman,) took place in this case in nine hours after her admission. The patient recovered without a bad symptom.

"Another was admitted in the eighth month of pregnancy; she was seized with labor the day before, and the waters had been discharged for four hours previous to her admission. No difficulty, however, was experienced in turning and the woman recovered rapidly."

The third arm case was a second child in an instance of twins.

"Of the crotchet cases.—The operation in one instance was had recourse to in consequence of deformed pelvis, and the length of time she had been in labour previous to her admission, besides which there were unequivocal evidence of the child's death. The operation was performed with ease, and the child extracted with perfect safety to the mother, but very unfavorable symptoms soon showed themselves, and ultimately sloughing of the neck of the uterus and adjoining parts took place, which caused her death fourteen days after delivery.

"Another was a soldier's wife, who had been in labour four days previous to her being sent into hospital, during which time she said she had not passed water: the bladder was greatly distended. The operation of embryotomy was had recourse to immediately after her admission; the head being so impacted in the pelvis as to prevent the use of the forceps. When the child was extracting, it was found to be very much enlarged from putrefaction: she lived for nearly three weeks, having labored under a continued hoarseness, and nearly loss of voice. These symptoms existed at the time of her admission. On examination after death, there was ulceration of the chordæ vocales, and evident inflammation of the mucous membrane of the bronchial tubes. The uterus had two small abscesses in its substance. The third was operated on in consequence of the length of time, eighteen hours, the head had been in the cavity of the pelvis, without the slightest advance, though the labour-pains continued violent, and after the forceps had been tried. This woman speedily recovered.

"Of the funis presentations.—Two cases occurred in women who had come a considerable distance on foot, from the country, to be confined; a large portion of the cord was down, and pulsation had ceased previous to admission.

"In the third case of funis presentation, the discharge of the waters and the falling down of the funis, were the first symptoms which attracted the patient's notice, the former having fallen considerably below the external parts without any previous pain.

"Of the footling cases, one was an acephalous fœtus, and the cord presented along with the foot. Of the breech presentations, three were cases of premature labour.

"The following is a brief account of the remaining seven cases of death which occurred:

"Mary Nowlan died of rheumatic fever a fortnight after delivery.

"Mary Cullen died of hemorrhage, occurring immediately after the expulsion of the child. Her death was almost instantaneous.

"Margaret Butler died in consequence of inflammation of the uterus and some of the adjacent parts.

"Judith Riley was extremely delicate on coming into hospital. Inflammation and sloughing of the upper and inner part of the uterus took place, which was not suspected during her lifetime; after death, on cutting into the diseased part, (which was not much greater in extent than the size of a crown piece,) it had much the characters of anthrax.

"Mary M'Dermot died of consumption three days after delivery, which she had been labouring under for a long period previous to her admission.

"Mary Maguire was admitted on the night of the 24th of July, 1830, and was delivered in two hours after. She was so ill from chest disease, apparently phthisis, that it was thought she could not survive the delivery. She died three hours afterwards; no examination was allowed.

"Judith Foy was labouring under bronchitis on her admission, which rapidly increased after her delivery: she sunk in two days.

"In one instance premature labour was induced in consequence of contracted pelvis, in a woman named Mary Fox, of diminutive stature. About fourteen hours after the evacuation of the liquor amnii, she had a severe rigor, which lasted for an hour. In twelve hours after, slight labour pains occurred; these continued for about 9 hours, when she was delivered. The child was born alive, but died in an hour.—*Dublin Hospital Reports, Vol. V.*

28. *Davy and Wollaston compared as Philosophers.*—In contrasting the genius of Wollaston with that of Davy, let me not be supposed to invite a comparison to the disparagement of either, but rather to the glory of both, for by mutual reflection each will grow the brighter. If the animating principle of Davy's mind was a powerful imagination, generalizing phenomena, and casting them into new combinations, so may the striking characteristic of Wollaston's genius be said to have been an almost superhuman perception of minute detail. Davy was ever imagining something greater than he knew; Wollaston always knew something more than he acknowledged:—in Wollaston the predominant principle was to avoid error; in Davy, it was the desire to discover truth. The tendency of Davy, on all occasions, was to raise probabilities into facts; while Wollaston as continually made them subservient to the expression of doubt.

Wollaston was deficient in imagination, and under no circumstances could he have become a poet nor was it to be expected that his investigations should have led him to any of those comprehensive generalizations which create new systems of Philosophy. He well knew the compass of his powers, and he pursued the only method by which they could be rendered available in advancing knowledge. He was a giant in strength, but it was the strength of Antæus, mighty only on the earth. The extreme caution and reserve of his manner were inseparably connected with the habits of his mind; they pervaded every part of his character; in his amusements, and in his scientific experiments, he displayed the same nice and punctilious observation—whether he was angling for trout, or testing for elements, he alike relied for success upon his subtile discrimination of minute circumstances.

By comparing the writings as well as the discoveries of these two great philosophers, we shall readily perceive the intellectual distinctions I have endeavored to establish. "From their fruits shall ye know them." The discoveries of Davy were the results of extensive views and new analogies; those of Wollaston were derived from a more exact examination of minute, and, to ordinary observers, scarcely appreciable differences. This is happily illustrated by a comparison of the means by which each discovered new metals. The alkaline basis were the products of a comprehensive investigation, which had developed a new order of principles; the detection of palladium and rhodium among the ores of platinum, was the reward of delicate manipulation and microscopic scrutiny. As chemical operators, I have already pointed out their striking peculiarities, and they will be found to be in strict keeping with the other features of their respective characters. I might extend the parallel farther; but Dr. Henry, in the eleventh edition of his system of Chemistry, has delineated the intellectual portraits of these two great philosophers with so masterly a hand, that, by referring to the passage, all farther observation will be rendered unnecessary.—*Boston Med. and Surg. Journ.*

29. *The Anatomy Bill.*—The subjoined is the Anatomy Bill as it has passed through all its stages in both branches of the Legislature of this State, and received the signature of the Governor. As it is now the law of the land, we have thought that the profession would desire to possess a correct copy, which they have below.

Commonwealth of Massachusetts. In the year of our Lord One Thousand Eight Hundred and Thirty-one.

*An Act more effectually to protect the Sepulchres of the Dead, and to legalize the Study of Anatomy in certain Cases.*

Sect. 1. *Be it enacted by the Senate and House of Representatives, in General Court assembled, and by the authority of the same,* That if any person, not being authorised by the Board of Health, Overseers of the Poor, or Selectmen, in any town of this Commonwealth, or by the Directors of the House of Industry, Overseers of the Poor, or Mayor and Alderman, of the City of Boston, in said Commonwealth, shall knowingly or wilfully dig up, remove, or convey away, or aid and assist in digging up, removing or conveying away, any human body, or the remains thereof,—such person or persons so offending, on conviction of such offence in the Supreme Judicial Court of this Commonwealth, shall be adjudged guilty of felony, and shall be punished by solitary imprisonment for a term not exceeding ten days, and by confinement afterwards to hard labor for a term not exceeding one year; or shall be punished by a fine not exceeding two thousand dollars to enure to the benefit of the Commonwealth, and by imprisonment in the common jail for a term not exceeding two years at the discretion of the Court, according to the nature and aggravation of the offence.

Sect. 2. *Be it further enacted,* That if any person shall be in any way, either before or after the fact, accessary to the commission, by any person or persons, of the offence described in the first section of this act, such person or persons shall be adjudged and taken to be principals, and shall be, on conviction of the Court aforesaid, subject to the same punishments and forfeitures as are in said first section provided.

Sect. 3. *Be it further enacted,* That from and after the passing of this act, it shall be lawful for the Board of Health, Overseers of the Poor, and Selectmen of any town in this Commonwealth, and for the Directors of the House of Industry, Overseers of the Poor, and Mayor and Aldermen, of the City of Boston, in said Commonwealth, to surrender the dead bodies of such persons, except town paupers, as may be required to be buried at the public expense, to any regular physician, cduly licnsed according to the laws of this Commonwealth—to be by said physician used for the advancement of anatomical science; preference being always given to the Medical Schools that now are or hereafter may be established by law in this Commonwealth, during such portions of the year as *such* schools, or either of them may require subjects for the instruction of Medical Students:—*Provided always*, That no such dead body shall in any case be so surrendered, if, within thirty-six hours from the time of its death, any one or more persons claiming to be kin, friend or acquaintance to the deceased, shall require to have said body inhumed; or, if it be made to appear to the Selectmen or Overseers of the Poor, of any town in this Commonwealth, or to the Mayor and Aldermen or Overseers of the Poor of the City of Boston, that such dead body is the remains of a stranger or traveller, who suddenly died before making known who or whence he was: but said dead body shall be inhumed, and, when so inhumed, any person disinterring the same for purposes of dissection, or being accessary, as is described in the second section of this act, to such exhumation, shall be liable to the punishments and forfeitures in this act respectively provided:—*And provided further*, That every physician so receiving any such dead body, before it be lawful to deliver him the same, shall in each case give to the Mayor and Aldermen of the city of Boston, or to the Selectmen of any town of this Commonwealth, as each case may require, good and sufficient bond or bonds, that each body by him so received, shall be used only for the promotion of anatomical science; that it shall be used for such purposes only in the Commonwealth, and so as in no event to outrage the public feeling; and that, after having so used, the remains thereof shall be decently inhumed.

Sect. 4. *Be it further enacted*, That from and after the passing of this act, it shall be lawful for any physician duly licensed according to the Laws of this Commonwealth, or for any medical student under the authority of any such physician, to have in his possession, to use and employ, human dead bodies, or the parts thereof, for purposes of anatomical inquiry and instruction.

Sect. 5. *Be it further enacted*, That nothing in this act shall be so construed, as to give to the Board of Health, Overseers of the Poor, or Selectmen of any town in this commonwealth, or to the Directors of the House of Industry, Overseers of the Poor, or Mayor and Aldermen of the City of Boston, in said Commonwealth, any power to license the digging up of any dead human body, or the remains thereof, other than was possessed by them before the passing of this act, or is given them by the third section of this act.

Sect. 6. *Be it further enacted*, That the act passed March 2, 1815, entitled " An Act to protect the Sepulchres of the Dead," and also all other Acts, or parts of Acts, contravening the provisions of this Act, be and the same are hereby repealed *Boston Med. and Surg. Journ.*

*Approved by the Governor, Feb.* 28.

